# Docetaxel-loaded pH/ROS dual-responsive nanoparticles with self-supplied ROS for inhibiting metastasis and enhancing immunotherapy of breast cancer

**DOI:** 10.1186/s12951-023-02013-y

**Published:** 2023-08-22

**Authors:** Yu Wang, Qianmei Wang, Xiaowen Wang, Pu Yao, Qing Dai, Xiaowei Qi, Ming Yang, Xiao Zhang, Rong Huang, Jing Yang, Qian Wang, Peiyuan Xia, Dinglin Zhang, Fengjun Sun

**Affiliations:** 1 Department of Pharmacy, Southwest Hospital, Army Medical University (Third Military Medical University), Chongqing, 400038 China; 2 Department of Chemistry, College of Basic Medicine, Army Medical University (Third Military Medical University), Chongqing, 400038 China; 3 Department of Breast and Thyroid Surgery, Southwest Hospital, Army Medical University (Third Military Medical University), Chongqing, 400038 China; 4 Department of Pharmacy, Affiliated Hospital of North Sichuan Medical College, Nanchong, 637000 China; 5 Department of Stem Cell and Regenerative Medicine, Southwest Hospital, Army Medical University (Third Military Medical University), Chongqing, 400038 China; 6 Department of Urology, Southwest Hospital, Third Military Medical University (Amy Medical University), Chongqing, 400038 China

**Keywords:** pH/ROS dual-responsive NPs, Immunogenic cell death, Chemotherapy, Immunotherapy, Breast cancer

## Abstract

**Background:**

Although stimuli-responsive nanoplatforms were developed to deliver immunogenic cell death (ICD) inducers to enhance cancer immunotherapy, the complete release of ICD inducers into the tumor microenvironment (TME) was limited by the inadequate supplementation of endogenous stimulus (e.g., reactive oxygen species (ROS)). To address this issue, we synthesized a self-responsive nanomaterial with self-supplied ROS, which mainly consists of a ROS responsive moiety HPAP and cinnamaldehyde (CA) as the ROS-generating agent. The endogenous ROS can accelerate the degradation of HPAP in materials to release docetaxel (DTX, an ICD inducer). In intracellular acidic environment, the pH-sensitive acetal was cleaved to release CA. The released CA in turn induces the generation of more ROS through mitochondrial damage, resulting in amplified DTX release. Using this self-cycling and self-responsive nanomaterial as a carrier, DTX-loaded pH/ROS dual-responsive nanoparticles (DTX/FA-CA-Oxi-αCD NPs) were fabricated and evaluated in vitro and in vivo.

**Results:**

In vitro experiments validated that the NPs could be effectively internalized by FA-overexpressed cells and completely release DTX in acidic and ROS microenvironments to induce ICD effect. These NPs significantly blocked 4T1 cell migration and decreased cell invasion. In vivo experiments demonstrated that the tumor-targeted NPs significantly inhibited tumor growth and blocked tumor metastasis. More importantly, these NPs significantly improved immunotherapy through triggering effector T-cell activation and relieving the immunosuppressive state of the TME.

**Conclusions:**

Our results demonstrated that DTX/FA-CA-Oxi-αCD NPs displayed great potential in preventing tumor metastasis, inhibiting tumor growth, and improving the efficacy of anti-PD-1antibody.

**Graphical Abstract:**

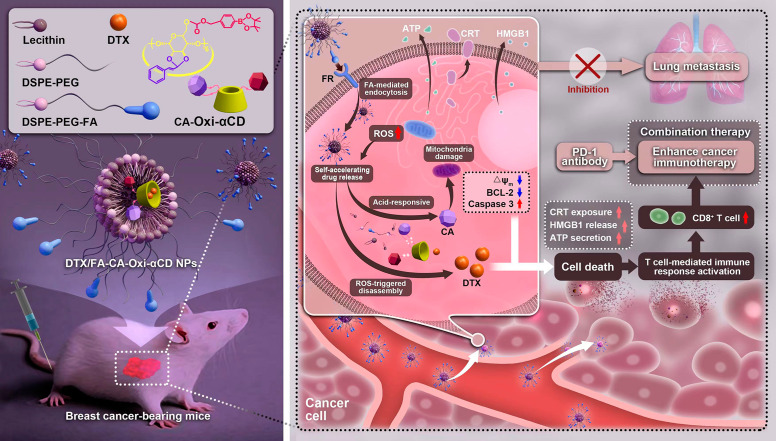

**Supplementary Information:**

The online version contains supplementary material available at 10.1186/s12951-023-02013-y.

## Background

Breast cancer is the most common malignant tumor in women worldwide with high morbidity and mortality, and its incidence is increasing rapidly in recent years [1]. Among them, triple-negative breast cancer (TNBC) accounts for 15-20% of all breast cancers, which is unresponsive to conventional treatment and has clinical features including high systemic metastasis, drug resistance, poor prognosis, and lower survival than other types of breast cancer [2–4]. It has been reported that about 30% of patients occur metastasis, which obviously increases the mortality rate of breast cancer [5]. Chemotherapy uses cytotoxic small molecules as the most commonly treatment against TNBC [6]. However, cytotoxic drugs caused severe side effects and had inadequate therapeutic efficacy. NPs-based drug delivery system (DDS) have drawn extensive attention in recent years due to their unique advantages, such as improving solubility, increasing drug accumulation in tumors, reducing side effects, and enhancing therapeutic efficacy of cytotoxic drugs [7–9]. A number of previous studies focused on the rational design to protect the encapsulated drugs and improve the stability of the NPs-based drug delivery system, but realizing efficient drug release from such stable system has become a new-born obstacle for clinic applications [10, 11]. To address this issue, stimuli-responsive nanoplatforms were developed to deliver anticancer drugs to enhance release ability of NPs and cancer therapy [12, 13].

Based on the abnormal physiological signals (e.g., weak acidity, high concentration of ROS, hypoxia, specific enzymes expression, etc.) in TME, smart responsive nanomedicines were exploited for targeting treatment of tumor [14]. For example, pH-responsive nanomedicines were developed for targeting therapy of breast cancer sine low pH values was detected in TME [15, 16]. Alternatively, excess ROS was observed in cancer cells, which plays a crucial role in development, progression, and treatment of breast cancer [17]. Consequently, smart release of anticancer drugs can be achieved based on the high concentration of ROS in breast cancer microenvironment [18–22]. However, complete release of payloads from vehicles at tumor site may be limited by insufficient endogenous ROS supplementation [23, 24]. Self-supplying ROS vehicles or pH/ROS dual responsive nanoplatform may overcome the mentioned deficiency and can be served as candidates for targeting treatment of breast cancer [25–28]. Moreover, TME-responsive nanomedicine have been developed for enhanced cancer immunotherapy [12, 13].

With the continuous exploration of the antitumor mechanism based on the immune system and immune characteristics, targeting and harnessing the intrinsic immune system for TNBC inhibition has shown tremendous promise. Various therapeutic approaches have been applied to activate antitumor immune responses, such as radiotherapy, photodynamic therapy, and chemotherapy [29–32]. In addition, a programmed death protein ligand 1 (PD-L1) inhibitor (e.g., PD1) has also been employed for immunotherapy of TNBC because the PD-L1 inhibitor can block the binding of PD1 on T cells with PD-L1 on tumor cells, which makes tumor cells reidentified and killed by T cells [33], but their efficacies are just passable [34]. The reason may be that the TME of TNBC is in an immunosuppressive state and shows a poor response to immunotherapy [35]. Therefore, regulation of the TME in TNBC has been widely investigated to improve the efficacy of immunotherapy. Recent studies have shown that ICD is a key strategy for converting “cold tumors” into immune-responsive tumors [36]. Importantly, some chemotherapeutics, such as DTX, doxorubicin, and cisplatin, can be used as ICD-inducing drugs [37]. These therapeutics can induce an ICD response, accompanied by the exposure of calreticulin (CRT) on dying cell surfaces and the release of damage-associated molecular patterns, including high mobility group Box 1 (HMGB1) and adenosine-5′-triphosphate (ATP), which can act as adjuncts to the innate immune system to create a favorable environment for immunotherapy [37]. In addition, immunotherapy can also improve the sensitivity of tumor cells to chemotherapeutics, reverse drug resistance, and reduce systemic toxicity, indicating that chemoimmunotherapy is a promising and convenient strategy for the treatment of TNBC.

DTX, a member of the taxane family and one of the most widely used cytotoxic drugs, was also reported to induce an ICD response in various cancer cells, including TNBC [38]. Thus, the combination of a DTX loaded pH/ROS dual-responsive nanoparticles and the use of immune checkpoint inhibitors could be promising synergistic chemoimmunotherapy.

**Fig. 1:**
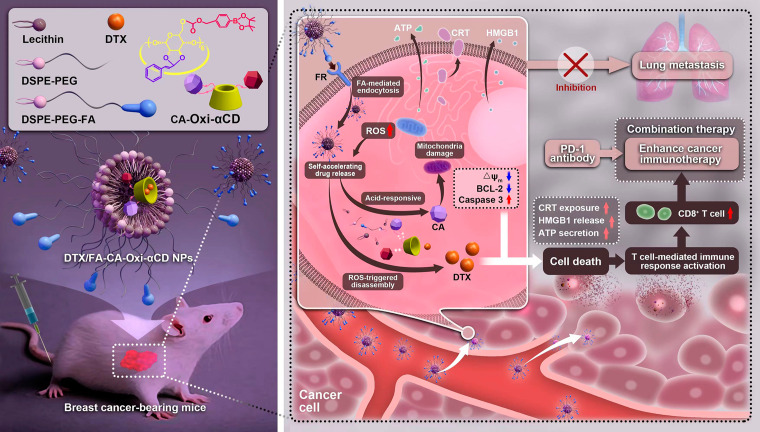
Schematic illustration of DTX-loaded pH/ROS dual-responsive nanoparticles combined chemotherapy and anti-PD-1 antibody to enhance immunotherapy of TNBC

Herein, pH/ROS dual-responsive materials were synthesized by conjugating CA and 4-hydroxymethylphenylboronic acid pinacol ester (HPAP, a ROS-responsive moiety) onto cyclodextrin (α-CD). CA, a primary component of cinnamon oil, can induce ROS generation and inhibit the growth of tumor cells with poor cytotoxicity [26]. The endogenous ROS can accelerate the degradation of HPAP in materials to release DTX. In addition, (HO)_3_B and H_2_CO_3_, the degradation products of HPAP, which can create an acidic microenvironment, cleaving the pH-sensitive acetal to release CA. The released CA in turn induces the generation of more ROS through mitochondrial damage, resulting in amplified DTX release. Using this self-cycling and self-responsive nanomaterial as a carrier, DTX-loaded pH/ROS dual-responsive NPs (DTX/CA-Oxi-αCD NPs) were fabricated through a nanoprecipitation/self-assembly method. The periphery of the NPs were modified with folic acid (FA) (DTX/FA-CA-Oxi-αCD NPs) because it exhibits a strong affinity for the folate receptor (FR), which is overexpressed in TNBC [39]. In vitro experiments indicated that the DTX/FA-CA-Oxi-αCD NPs were efficiently internalized by FR-overexpressed 4T1 cells and that accelerated DTX release profiles from the internalized NPs were observed under the pH/ROS conditions. DTX exerts its cytotoxicity to kill tumor cells and then induces the ICD effect. In vivo experiments revealed that DTX/FA-CA-Oxi-αCD NPs effectively accumulated at the 4T1 tumor site, and the released DTX significantly blocked tumor growth, inhibited tumor metastasis, and enhanced immunotherapy. Our studies here using the DTX/FA-CA-Oxi-αCD NPs combined with anti-PD-1 antibody to provide a candidate for targeted treatment of TNBC by chemotherapy combined with immunotherapy (Fig. 1).

## Materials and methods

### Reagents

The Dulbecco’s modified Eagle’s medium (DMEM) and fetal bovine serum were obtained from HyClone Inc. (Waltham, MA, USA). The streptomycin-penicillin solution was provided by Solarbio Life Sciences Co., Ltd. (Beijing, China). The 4’,6-Diamidino-2-phenylindole (DAPI), Hydrogen Peroxide Assay Kit, Enhanced Cell Counting Kit-8, and Mitochondrial membrane potential assay kit with JC-1 were supplied by Beyotime Biotechnology Co., Ltd. (Shanghai, China). The matrigel was purchased from Corning Inc. (Corning, NY, USA). The anti-mouse anti-PD-1 antibody was obtained from Bio X Cell (CD279, West Lebanon, New Hampshire, USA).

### Cells

The mouse breast cancer cell line 4T1 and human breast cancer cell line MDA-MB-231 were obtained from the Cell Bank of the Chinese Academy of Sciences (Shanghai, China). The 4T1 and MDA-MB-231 cells were incubated in a DMEM cell culture medium supplemented with 10% fetal bovine serum, 100 µg/mL streptomycin, and 100 IU penicillin at 37 °C in a humidified atmosphere containing 5% CO_2_.

### Animals

Six- or eight-week-old female BAlB/c mice or Kunming mice weighing approximately 20 g were supplied from the experimental animal center of Army Medical University (Chongqing, China) and kept in an SPF-level sterile animal room.

### Fabrication and characterization of DTX-encapsulated NPs

The Lecithin (6 mg) and DSPE-PEG_2000_ (6 mg) were dissolved in ethanol and Deionized water, and the solution was heated at 65℃ for 30 min and then cooled to room temperature naturally. CA-Oxi-αCD (50 mg) and DTX (10 mg) were dissolved in methanol and DMSO, and the drug-containing solution was added dropwise to the lipid dispersed solution with gentle stirring. After quickly stirred for 3 min, the mixture was self-assembled at room temperature for 2 h under slow stirring. Subsequently, the mixture was centrifuged at 15,000 × g for 10 min, washed with 5% F127, and finally suspended in ultrapure water. The preparation of the DTX/FA-CA-Oxi-αCD NPs was performed using the same procedure as that used for the NPs with slight changes: DSPE-PEG_2000_ (4 mg) and FA-PEG_3400_-DSPE (4 mg) were added to the lipid dispersion. The Blank FA-CA-Oxi-αCD NPs were fabricated without the addition of DTX, and Cy5-conjugated α-CD (5 mg) was utilized to fabricate the Cy5-CA-Oxi-αCD NPs and Cy5-FA-CA-Oxi-αCD NPs. In addition, the preparation of Oxi-αCD NPs and PLGA NPs was described in our previous study [19].

The size, polydispersity index (PDI), and zeta potential of the NPs were determined by dynamic light scattering (DLS) and laser Doppler anemometry using a Malvern Zetasizer (Nano ZS, Malvern, UK). The morphology of the NPs was imaged by transmission electron microscope (TEM, JEM-1400, Tokyo, Japan). The FT-IR spectra of α-CD, CA-αCD, and CA-Oxi-αCD were recorded on a PerkinElmer FT-IR spectrometer (100 S). To check the stability of the NPs, the freshly prepared NPs were diluted in ultrapure water or 10% fetal bovine serum (FBS), followed by the measurement of size and PDI distribution at interval time points using a Malvern Zetasizer (Nano ZS, Malvern, UK).

To investigate the responsiveness of the DTX/FA-CA-Oxi-αCD NPs under pH/H_2_O_2_ conditions, the NPs were incubated with ddH_2_O, pH 5.0, 1 mM H_2_O_2_, and pH 5.0/1 mM H_2_O_2_ for 2 h, respectively. Then the morphological changes of the NPs were observed by TEM.

The drug loading (DL) and encapsulation efficiency (EE) of DTX in the DTX/CA-Oxi-αCD NPs and DTX/FA-CA-Oxi-αCD NPs were calculated according to the following equations:

$$\begin{array}{l}{\rm{DL\% }}\,{\rm{ = }}\,\left( {{\rm{Amount}}\,{\rm{of}}\,{\rm{DTX}}\,{\rm{in}}\,{\rm{NPs/Weight}}\,{\rm{of}}\,{\rm{NPs}}} \right)\,\\{\rm{ \times 100\% }}{\rm{.}}\end{array}$$

$$\begin{array}{l}{\rm{EE\% }}\,{\rm{ = }}\,\left( {{\rm{Amount}}\,{\rm{of}}\,{\rm{DTX}}\,{\rm{in}}\,{\rm{NPs/Weight}}\,{\rm{of}}\,{\rm{feeding}}\,{\rm{DTX}}} \right)\,\\{\rm{ \times 100\% }}{\rm{.}}\end{array}$$

### Evaluation of pH/ROS-triggered disassembly of NPs

60 µL of freshly prepared NPs was co-incubated with PBS, 1 mM H_2_O_2_ in PBS, PBS at pH 5.0, or 1 mM H_2_O_2_ in PBS at pH 5.0 at 37 °C with gently shaking at 200 rpm. At the determined time point (0 h, 0.083 h, 0.25 h, 0.5 h, 1 h, 2 h, 4 h, 6 h, 8 h, 12 h, 24 h), the samples were taken out, and imaged to observe the disassembly of the NPs in the different medium.

In another experiment, 120 µL of freshly prepared NPs were co-incubated with different media as described above under the same conditions. In contrast, at the determined time points, the absorbance of the samples was determined by a Spectrum Instruments SP-721E Spectrophotometer.

### Drug release in vitro

The DTX and CA release behavior from the DTX/CA-Oxi-αCD NPs and DTX/FA-CA-Oxi-αCD NPs in vitro were investigated by the dialysis method. Briefly, 200 µL of the NPs (containing 2.88 mg DTX and 0.6 mg CA) were transferred into a dialysis bag (MWCO: 3500 Da), which was then immersed into 40 mL of PBS, 1 mM H_2_O_2_ in PBS, PBS at pH 5.0, or 1 mM H_2_O_2_ in PBS at pH 5.0. Both release media contained 1% (w/v) Tween 80. Then, these dialysis bags were placed at 37 °C with gently shaking at 100 rpm. At predetermined time points, including 0.5 h, 1 h, 2 h, 4 h, 6 h, 8 h, 10 h, 12 h, 24 and 48 h, 4.0 mL of external release medium was removed and replaced with the same volume of corresponding fresh medium. The concentrations of DTX and CA at each time point were determined by high performance liquid chromatography (HPLC, Waters, Milford, MA, USA) and the cumulative drug release percentage was calculated accordingly. The DTX release behavior from the DTX/PLGA NPs in vitro was investigated as above described.

### Hemolysis assay

The hemolysis assay was conducted by incubating the NPs with freshly isolated sheep blood cells following our previously published method [19]. Briefly, 3% red blood cell suspensions were incubated with different concentrations of various NPs for 1 h at 37 °C in 5% CO_2_. PBS was used as the negative control, and the positive control was a 1% *w*/*v* solution of Triton X-100. The supernatant absorbance was measured by Thermo Multiskan Spectrum spectrophotometer (Thermo Scientific Inc. Waltham, MA, USA) at the wavelength of 450 nm after centrifugation at 3000 rpm for 5 min. The hemolytic percentage (hemolysis %) was calculated according to the following equation: Hemolysis % = [A_450_ (NPs) – A_450_ (PBS)]/[A_450_ (1% Triton X-100) – A_450_ (PBS)] ×100%. All hemolysis experiments were carried out in triplicate.

### In vitro cytotoxicity assays

The in vitro cytotoxicity of DTX, CA, or nanoformulations against 4T1 cells and MDA-MB-231 cells were investigated by the CCK-8 assay. The cells were cultured in 96-well plates at 1 × 10^4^ cells per well for 24 h. Then, the cells were treated with DTX, CA, or nanoformulations at various concentrations (0.625, 1.25, 5, 10, 20, 40 ng/mL) for 48 h. The cells treated with a cell culture medium were used as a control. Finally, the absorbance of the cultures was detected at 450 nm using a Thermo Multiskan Spectrum spectrophotometer (Thermo Scientific Inc. Waltham, MA, USA).

### In vitro cell migration assay and the evaluation of cell invasion

The wound healing assay was used to visually observe and qualitatively evaluate cell migration. Briefly, 4T1 cells were seeded into 6-well culture plates at a density of 3 × 10^5^ cells per well. After the cells grew to 95%, the cell monolayers were wounded with sterile plastic pipette tips and carefully washed three times with PBS to remove floating cells. Subsequently, the cells were incubated with DTX, Blank NPs, DTX/CA-Oxi-αCD NPs, and DTX/FA-CA-Oxi-αCD NPs at an equivalent concentration of 20 ng/mL DTX for 24 or 48 h. Finally, the cell wound was imaged with an inverted fluorescence microscope (Olympus, IX-71, Tokyo, Japan) at 0, 24 and 48 h.

The 24-well Transwell plates with 8 μm pore polycarbonate filters (3422, Corning, NY, USA) were used to evaluate the inhibitory ability of the NPs on 4T1 cell invasion. First, the Transwell plates were coated with 2.67 mg/mL Matrigel and incubated at 37 °C overnight. Then, 4T1 cells were seeded into the upper chamber, and the lower chamber was filled with 700 µL of serum-containing DMEM. After the cells were allowed to adhere for 24 h, they were incubated with DTX, Blank NPs, DTX/CA-Oxi-αCD NPs, and DTX/FA-CA-Oxi-αCD NPs at a final concentration of 20 ng/mL DTX in serum-free DMEM for 48 h. Then, the cells remaining on the upper side of the Transwell membrane were carefully wiped away with cotton swabs, and the cells on the lower side were fixed with 4% paraformaldehyde for 10 min. Afterward, 0.1% crystal violet solution was used to stain the cells on the bottom side for 10 min, followed by washing and drying of the chamber. An inverted fluorescence microscope (Olympus, IX-71) was used to photograph the cells. Finally, the crystal violet in the stained cells was dissolved with 30% (*v*/*v*) acetic acid aqueous solution and then measured at 570 nm using a Thermo Multiskan Spectrum spectrophotometer (Thermo Scientific Inc. Waltham, MA, USA).

### Cell uptake and intracellular drug release of the NPs

4T1 and MDA-MB-231 cells were cultured in confocal dishes. After the cells adhered for 24 h, the cells were treated with free Cy5, Cy5-labeled CA-Oxi-αCD NPs, or Cy5-labeled FA-CA-Oxi-αCD NPs at a final concentration of 2 µg/mL for 6 h. At the determined time, the cells were washed with ice-cold PBS three times and then fixed with 4% paraformaldehyde at room temperature. Then, the cell nuclei were stained with DAPI for 10 min. Finally, the cellular uptake efficiency of the NPs was observed by confocal laser scanning microscopy (CLSM, Carl Zeiss, Baden-Württemberg, Germany).

To confirm the active targeting capability of the FA-modified NPs to the 4T1 cells, the FA receptor on the surface of the 4T1 cells was pre-blocked with FRα antibodies (1:500, DF4058, Affinity Biosciences, OH, NJ, USA) for 2 h after being cultured in 20 mm confocal dishes for 24 h. Then, the cells were treated with free Cy5, Cy5-labeled CA-Oxi-αCD NPs, or Cy5-labeled FA-CA-Oxi-αCD NPs for 6 h. Next, the cell nuclei were stained with DAPI for 10 min. Finally, the cell uptake efficiency was imaged using the CLSM (Carl Zeiss, Baden-Württemberg, Germany).

The intracellular drug release of NPs was also determined by LC-MS/MS (AB SCIEX Qtrap5500, Shimadzu, Kyoto, Japan). Briefly, the 4T1 cells were cultured in a 25 mm^2^ cell culture flask for 24 h and then treated with DTX, Blank NPs, DTX/CA-Oxi-αCD NPs, or DTX/FA-CA-Oxi-αCD NPs at a final concentration of DTX of 20 ng/mL for 48 h. Cells were treated with cell culture medium as the control. Then, the cells were collected and repeatedly frozen and thawed. Finally, the intracellular concentration of DTX released from the NPs was determined by LC-MS/MS (AB SCIEX Qtrap5500, Shimadzu, Kyoto, Japan).

### The penetration of the NPs in tumor spheroids

First, confocal dishes were precoated with a thin layer of Matrigel. Then, 2 × 10^5^ 4T1 cells were seeded on confocal dishes for 48 h at 37 °C with 5% CO_2_. Then, the tumor spheroids were treated with Cy5, Cy5-labeled CA-Oxi-αCD NPs, or Cy5-labeled FA-CA-Oxi-αCD NPs for 2 and 6 h. After treatment, the cells were washed with ice-cold PBS and fixed in 4% paraformaldehyde, and the cell nuclei were stained with DAPI for 10 min. Finally, the distribution of NPs in the 3D tumor spheroids was observed with the CLSM (Carl Zeiss, Baden-Württemberg, Germany) in layer scanning mode.

### Determination of intracellular H_2_O_2_ generation

4T1 cells were seeded at 2 × 10^5^ cells per well in 12-well plates and cultured for 24 h. Then, the cells were incubated with CA, PLGA NPs, Oxi-αCD NPs or CA-Oxi-αCD NPs for 48 h. Then, the intracellular concentrations of H_2_O_2_ were detected using a Hydrogen Peroxide Assay Kit according to the manufacturer’s instructions.

### Mitochondrial membrane potential assay

A JC-1 probe was employed to evaluate mitochondrial depolarization in 4T1 cells. Briefly, 4T1 cells or MDA-MB-231 cells were cultured in a 20 mm confocal dish for 24 h. Then, the cells were incubated with DTX, Blank NPs, DTX/CA-Oxi-αCD NPs, or DTX/FA-CA-Oxi-αCD NPs at an equivalent concentration of 10 ng/mL DTX for 48 h. Cells treated with cell medium served as a control. Then, the cells were incubated with an equal volume of serum-free medium containing JC-1 dye at 37 °C for 20 min, washed twice with PBS, and placed in a fresh medium without serum. Finally, mitochondrial depolarization in the 4T1 cells or MDA-MB-231 cells was determined by the CLSM imaging (Carl Zeiss, Baden-Württemberg, Germany). The images were observed at 490 nm excitation and 530 nm emission for green (JC-1 monomers) and 525 nm excitation and 590 nm emission for red fluorescence (JC-1 aggregates).

### Western blot analysis

4T1 cells were cultured in T25 cell flasks for 24 h at 37 °C in 5% CO_2_. Then, the cells were treated with DTX, Blank NPs, DTX/CA-Oxi-αCD NPs, or DTX/FA-CA-Oxi-αCD NPs at an equivalent concentration of 20 ng/mL DTX for 48 h, and the control was treated with cell culture medium. After 48 h, the cells were lysed and centrifuged, and the supernatant concentration was detected with a BCA kit (Beyotime Biotechnology Co., Ltd., Shanghai, China). The expression of cleaved-caspase-3 and phosphorylated Bcl-2 were analyzed by Western blot assay. In addition, the expression of the FA receptor on the surface of the 4T1 and MDA-MB-231 cells was also determined by Western blot analysis. The following antibodies were used for immunoblotting: rabbit monoclonal anti-cleaved-caspase-3 antibody (1:1000 dilution, Cell Signaling Technology, Inc. MA, USA), rabbit monoclonal anti-p-Bcl-2 antibody (1:1000 dilution, Cell Signaling Technology, Inc., Danvers, MA, USA), and rabbit monoclonal GAPDH antibody (1:1000 dilution, Cell Signaling Technology, Inc., Danvers, MA, USA), and FRα antibody (1:500, DF4058, Affinity Biosciences, OH, NJ, USA).

### Immunogenic cell death analysis

For the HMGB1 release and ATP determination assay, the 4T1 cells were seeded in 6-well plates to grow for 24 h. Then, the cells were treated with DTX, Blank NPs, DTX/CA-Oxi-αCD NPs, or DTX/FA-CA-Oxi-αCD NPs at an equivalent concentration of 10 ng/mL DTX, and the control was treated with a cell culture medium. After incubation for 48 h, the HMGB1 release and ATP level of the 4T1 cells were determined with an ELISA kit (Elabscience Biotechnology Co., Ltd., Wuhan, China) and an ATP Assay Kit (Beyotime Biotechnology Co., Ltd., Shanghai, China), respectively, according to the manufacturer’s instructions.

For the CRT exposure study, the 4T1 cells were cultured in a 20 mm confocal dish for 24 h. Then, the cells were incubated with DTX, Blank NPs, DTX/CA-Oxi-αCD NPs, or DTX/FA-CA-Oxi-αCD NPs at an equivalent concentration of 10 ng/mL DTX for 48 h. Cells treated with cell medium served as a control. After 48 h, the cells were fixed with 4% paraformaldehyde for 15 min, permeabilized with 0.5% Triton X-100 for 10 min, and blocked with 5% goat serum for 0.5 h. Then, the cells were incubated with a primary anti-CRT antibody (calreticulin (D3E6) XP® rabbit mAb, 1:1000, 12,238, Cell Signaling Technology, Inc., Danvers, MA, USA) at 4 °C overnight, washed three times with PBS, and incubated with a secondary antibody (Alexa Fluor 488-labeled goat anti-rabbit IgG-H&L, 1:1000, ab150077, Abcam, Cambridge, UK) for 2 h at room temperature in the dark. Cell nuclei were stained with DAPI for 10 min in the dark. Finally, the treated cells were observed by the CLSM (Carl Zeiss, Baden-Württemberg, Germany).

### Biodistribution assay by IVIS study

The in vivo distribution assay was performed in female BALB/c mice bearing 4T1 mammary tumors, which were injected with 100 µL of 4T1 cells and Matrigel cold mixture suspension (1 × 10^7^ cells/mL) into the fourth mammary fat pad (right). When the tumor volume grew to approximately 300 mm^3^, Cy5, Cy5-labeled CA-Oxi-αCD NPs, or Cy5-labeled FA-CA-Oxi-αCD NPs were injected intravenously at a Cy5 dosage of 1 mg/kg. At the determined time after administration, mice were anesthetized, and in vivo distribution images were obtained using an in vivo imaging system (Perkin Elmer, Waltham, MA, USA). At 24 and 48 h post-injection, the mice were sacrificed, and the tumors and major organs were collected and imaged. The fluorescence intensity within tumor tissues was correspondingly analyzed.

In addition, the tumor tissues were cryosectioned into 10 μm sections and fixed with 4% paraformaldehyde, and the cells nuclei were then stained with DAPI. The distribution of the NPs in the tumor tissues was obtained using the CLSM (Carl Zeiss, Baden-Württemberg, Germany).

### In vivo antitumor efficacy and survival curves

The in vivo breast tumor growth and spontaneous lung metastasis model was established by subcutaneous injecting 0.1 mL of 5 × 10^6^ 4T1 cells into the fourth mammary fat pad (right) of six-week-old female BALB/c mice. Primary tumor volumes were measured and calculated by the following formula: V = 1/2 (a × b^2^), in which a is the longest diameter and b is the shortest diameter perpendicular to the length. When the tumor volume grew to approximately 50 mm^3^, tumor-bearing mice were randomly divided into 6 groups and intravenously injected with PBS, DTX, Blank NPs, PLGA NPs, DTX/CA-Oxi-αCD NPs, or DTX/FA-CA-Oxi-αCD NPs at a dose of 5 mg/kg DTX every 4 days four consecutive times. The tumor volumes and body weights were measured every 2 days. On Day 22 and Day 29, all mice were sacrificed, and the tumors and major organs were harvested for imaging, weighed, and fixed in 4% paraformaldehyde for histological examination and immunohistochemistry/immunofluorescence examination. To quantify the lung metastatic burden, the lungs were intratracheally injected with 0.8 mL India ink and then fixed in Fekete’s solution. The number of metastatic nodules in each lung lobe was counted.

The remaining tissue was homogenized, and the DTX concentration in the major organs and tumor tissues was determined by LC-MS/MS (AB SCIEX Qtrap5500, Shimadzu, Kyoto, Japan).

The survival rate was also determined to evaluate the therapeutic efficacy of the NPs. Briefly, mice with a tumor volume of approximately 50 mm^3^ were randomly divided into 6 groups and then subjected to the same treatment as described above. The overall survival of the mice in each treatment group was analyzed by Kaplan-Meier curves. Data including the primary tumor diameter (> 2 cm) and natural death were recorded as a survival curve.

### Evaluation of pulmonary metastasis suppression

Female BALB/c mice were intravenously injected with 1 × 10^6^ 4T1-Luc cells at Day 0 (n = 5). After 24 h, PBS, Blank NPs, DTX, DTX/CA-Oxi-αCD NPs, or DTX/FA-CA-Oxi-αCD NPs at a DTX dose of 5 mg/kg were intravenously administered every 4 days five consecutive times. Mice that received PBS served as the control. Tumor progression was monitored weekly by an in vivo IVIS spectrum imaging system (PerkinElmer, Waltham, MA, USA). On Day 22, 0.2 mL of 15 mg/mL D-luciferin was administered via intraperitoneal injection to all mice, and bioluminescent images were obtained with an IVIS Spectrum Imaging System within 10 min. Then, the mice were quickly sacrificed, the major tissues were collected, and ex vivo bioluminescence images were captured. Subsequently, the lungs were fixed with Bouin’s fixative solution, then imaged and sectioned, and stained with H&E to observe the pulmonary metastatic nodules.

### Chemotherapy and immunotherapy combination treatment

We also investigated the antitumor effects of the NPs combined with anti-PD-1 antibody in the 4T1 tumor-bearing mice. Briefly, the 4T1 tumor-bearing mouse model was established as previously described. When the tumor volume grew to approximately 50 mm^3^, the tumor-bearing mice were randomly divided into 4 groups (n = 5). Mice were treated with 100 µL of DPBS intravenously, anti-mouse anti-PD-1 antibody i.p. injection at a 10 mg/kg dose, DTX/FA-CA-Oxi-αCD NPs via the tail vein at 5 mg/kg DTX, and DTX/FA-CA-Oxi-αCD NPs with anti-PD-1 antibody. For therapeutic efficacy studies, the DTX/FA-CA-Oxi-αCD NPs and anti-PD-1 antibodies were administered once every 4 days five consecutive times. The tumor volumes and body weights were measured every 2 days. After the indicated treatments, all the mice were sacrificed, and the tumors and spleens of the mice were isolated, ground up, and passed through a cell strainer to harvest single-cell suspensions. For analysis of T-cell activation in tumors and spleens, the collected cells were stained with anti-CD8-perCP-Cy5.5 (Biolegend, San Diego, CA, USA), anti-CD4-FITC (Biolegend, San Diego, CA, USA), and anti-CD3-PE-Cy7 (Biolegend, San Diego, CA, USA) and then detected using flow cytometry. Immunohistochemical staining was used to analyze the presence of CD4^+^ T cells and CD8^+^ T cells in the tumor sections.

### In vivo safety evaluation

The female Kunming mice (approximately 30 g) were randomly divided into 4 groups (n = 4) to evaluate the in vivo safety of the NPs. The control group received 0.1 mL of saline via the tail vein. In parallel, another group was intravenously injected with doses of 125, 250, and 500 mg/kg NPs. Mice were weighed, and their behaviors were observed every two days. On Day 15, all mice were sacrificed. Blood samples were collected for hematological analysis (Sysmex KX-21, Sysmex Co., Kobe, Japan). Major organs, including the heart, liver, spleen, lung, and kidneys, were collected, weighed and stained with H&E.

### Statistical analysis

All data are presented as the mean ± standard deviation (SD) of at least three independent experiments. Statistical analysis was performed using one-way variance (ANOVA) for more than three groups and the Student’s *t-test* for two groups. Statistical significance was defined as * *P* < 0.05, ** *P* < 0.01, ***P < 0.001 and ****P < 0.0001.

## Results and discussions

### Synthesis and characterization of pH/ROS dual-responsive materials

**Fig. 2:**
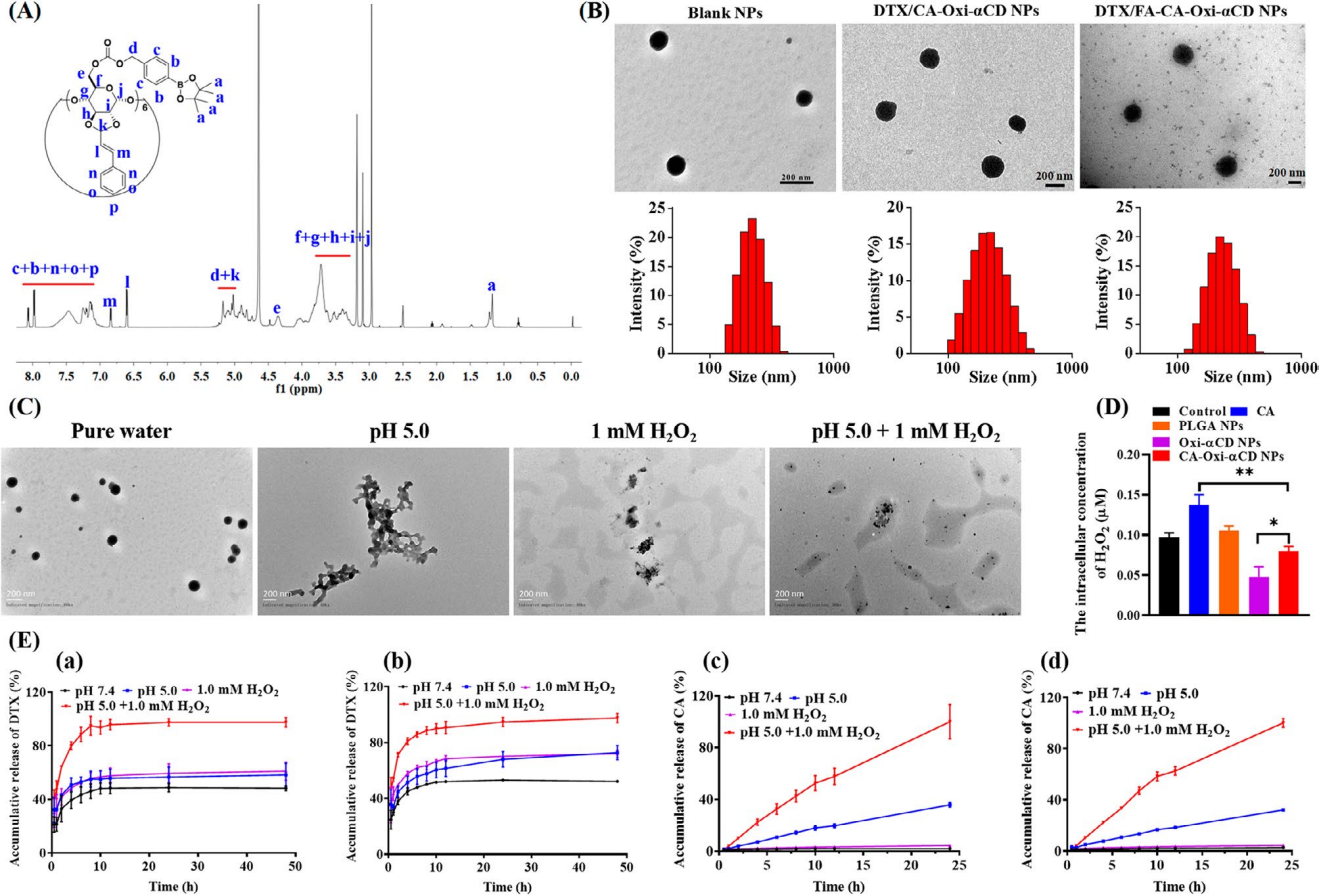
^1^H NMR spectrum of CA-Oxi-αCD carrier and the characterization of DTX/CA-Oxi-αCD NPs and DTX/FA-CA-Oxi-αCD NPs. (**A**) ^1^H NMR spectrum of CA-Oxi-αCD carrier. (**B**) Size distribution and morphology of Blank NPs, DTX/CA-Oxi-αCD NPs and DTX/FA-CA-Oxi-αCD NPs. (**C**) The change of morphology of DTX/FA-CA-Oxi-αCD NPs in various medium. (**D**) The intracellular concentration of H_2_O_2_ in 4T1 cells after different treatment. The controls were treated with cell culture medium. *p < 0.05, **p < 0.01, compared with CA-Oxi-αCD NPs (n = 3). (**E**) The in vitro drug release behavior of DTX/CA-Oxi-αCD NPs (a,c) and DTX/FA-CA-Oxi-αCD NPs (b,d) in various release medium for 48 h. (a,b) The released DTX. (c,d) The released CA. Data represent mean ± SD (n = 3)

The pH/ROS dual-responsive materials (CA-Oxi-αCD) were synthesized by conjugating CA and HPAP onto α-CD (Scheme S1, Supporting information). CA was conjugated onto α-CD by an acetal bond, and then CDI-activated HPAP was conjugated onto CA-αCD to obtain CA-Oxi-αCD materials. The ^1^ H NMR spectrum showed significant proton signals corresponding to the phenyl groups of CA and HPAP (~ 7.15 - ~8.20 ppm) (Fig. 2A, Figure S1 A(a,b)), indicating that CA and HPAP were successfully conjugated onto α-CD. In addition, the proton signals at ~ 6.70 and 6.90 ppm were attributed to the double bond of CA, and the signals at ~ 5.02 ppm indicated the presence of the CA acetal proton. The protons of α-CD appeared at ~ 3.20 - ~4.10 and ~ 4.35 ppm. The peaks at ~ 1.17 ppm and ~ 5.17 ppm were assigned to the methyl proton and benzyl methyl proton of HPAP, respectively. The FT-IR spectrum revealed absorption peaks relevant to carbonyl (1749 cm^− 1^), carbon-carbon double bonds (1672 cm^− 1^), and boron-oxygen bonds (1249 cm^− 1^) in the materials (Figure S1 A(c)), further demonstrating that CA and HPAP were successfully conjugated onto α-CD.

### Fabrication and characterization of the NPs

As summarized in Table S1, DLS analysis revealed that the average diameters of the DTX/CA-Oxi-αCD NPs and DTX/FA-CA-Oxi-αCD NPs were 213.9 ± 4.8 nm and 227.9 ± 0.8 nm, respectively. The NPs had a negative zeta potential, and had good dispersity according to their polymer dispersity index (PDI) were less than 0.2 (Fig. 2B). In addition, the FA modification and DTX loading did not significantly change the size, PDI, and the zeta potential of NPs. Additionally, TEM showed an accordant size with DLS characterization and confirmed the spherical morphology of the DTX/CA-Oxi-αCD NPs and DTX/FA-CA-Oxi-αCD NPs (Fig. 2B). The degradation of the Blank NPs in various media was also investigated. As shown in Figures S1B and S1C, the NPs retained stability after 24 h of PBS incubation. In contrast, about 80% of the NPs were degraded in the pH 5.0 or 1.0 mM H_2_O_2_ medium after 24 h of incubation (Figure S1 B and S1 C). The degradation of the NPs reached 100% in the pH 5.0/1.0 mM H_2_O_2_ medium with 6 h of incubation (Figure S1 B and S1 C). The morphology of the DTX-loaded NPs in various media after 2 h of incubation was also observed by TEM. TEM images revealed that the spherical morphology of the NPs collapsed to different degrees under acid and/or ROS medium treatment (Fig. 2C). The size distribution of the NPs was determined in a 10% FBS solution to evaluate the stability. DLS characterization demonstrated that there was no significant variation in the particle size of the DTX/CA-Oxi-αCD NPs and DTX/FA-CA-Oxi-αCD NPs within 48 h of incubation, which indicated that the NPs had good physiological stability in 10% FBS (Figure S1 D). In addition, both the DTX/CA-Oxi-αCD NPs and DTX/FA-CA-Oxi-αCD NPs exhibited good blood compatibility (Figure S2).

The DTX loading in the DTX/CA-Oxi-αCD NPs and DTX/FA-CA-Oxi-αCD NPs was 16.95 ± 3.07% and 21.53 ± 3.12%, respectively. The encapsulation efficiency of DTX in the DTX/CA-Oxi-αCD NPs and DTX/FA-CA-Oxi-αCD NPs was 59.34 ± 5.40% and 75.98 ± 6.90%, respectively. Compared to our reported ROS-responsive NPs, higher drug loading was obtained [40]. Introducing CA into Oxi-αCD may alter the affinity of the drug and materials.

To further investigate the effect of CA-Oxi-αCD NPs on intracellular ROS levels in 4T1 cells, the concentration of intracellular H_2_O_2_ was detected with different treatment. As shown in Fig. 2D, compared to the control group, the intracellular H_2_O_2_ concentration increased significantly when cells were treated with the CA, indicating that CA induced the generation of H_2_O_2_. And the intracellular H_2_O_2_ concentration in 4T1 cells was not affected by the non-responsive PLGA NPs, while the ROS-responsive Oxi-αCD NPs exhausted the intracellular H_2_O_2_ dramatically compared to the control. Importantly, the pH/ROS dual-responsive CA-Oxi-αCD NPs induced obviously higher H_2_O_2_ in 4T1 cells than Oxi-αCD NPs. The results indicated that acidic microenvironment in tumor cells cleaved the pH-sensitive acetal of CA-Oxi-αCD materials to release CA, which subsequently increased intracellular ROS concentration (Fig. 2D, Scheme S2).

Our previous experiments demonstrated that the pH/ROS dual-responsive nanovehicles rapidly collapsed under acid and/or ROS conditions. Therefore, the drug release profiles of the DTX-loaded NPs were investigated in acidic and/or ROS medium. As shown in Fig. 2E(a), approximately 40% of DTX was released from the DTX/CA-Oxi-αCD NPs within 48 h under neutral conditions (pH 7.4). Interestingly, almost all the DTX was released from the NPs with pH 5.0/1.0 mM H_2_O_2_ incubation, indicating that the drug release profiles were dramatically magnified under acidic and ROS conditions. Similar drug release profiles were observed in the DTX/FA-CA-Oxi-αCD NPs (Fig. 2E(b)). For non-responsive PLGA NPs, the DTX release profile from PLGA NPs displayed no significant difference in various medium (Figure S3). The release of CA from the DTX/CA-Oxi-αCD NPs was also studied. As shown in Fig. 2E(c), CA was almost undetectable in the PBS (pH 7.4) or H_2_O_2_ medium after 24 h of incubation. In contrast, the release of CA from the NPs gradually increased in the acidic medium (pH 5.0) within the experimental period. CA forms an acetal bond with α-CD, which can be cleaved under weakly acidic conditions. Importantly, the release of CA from the NPs was significantly accelerated in the pH 5.0/1.0 mM H_2_O_2_ medium (Fig. 2E(c)). HPAP can be degraded in the H_2_O_2_ medium to produce (HO)_3_B and H_2_CO_3_, which can create acidic conditions to facilitate further CA release from materials (Scheme S2). The CA release behavior from the DTX/FA-CA-Oxi-αCD NPs exhibited similar trends to that of the DTX/CA-Oxi-αCD NPs (Fig. 2E(d)).

### Cellular uptake and penetration into tumor spheroids

**Fig. 3:**
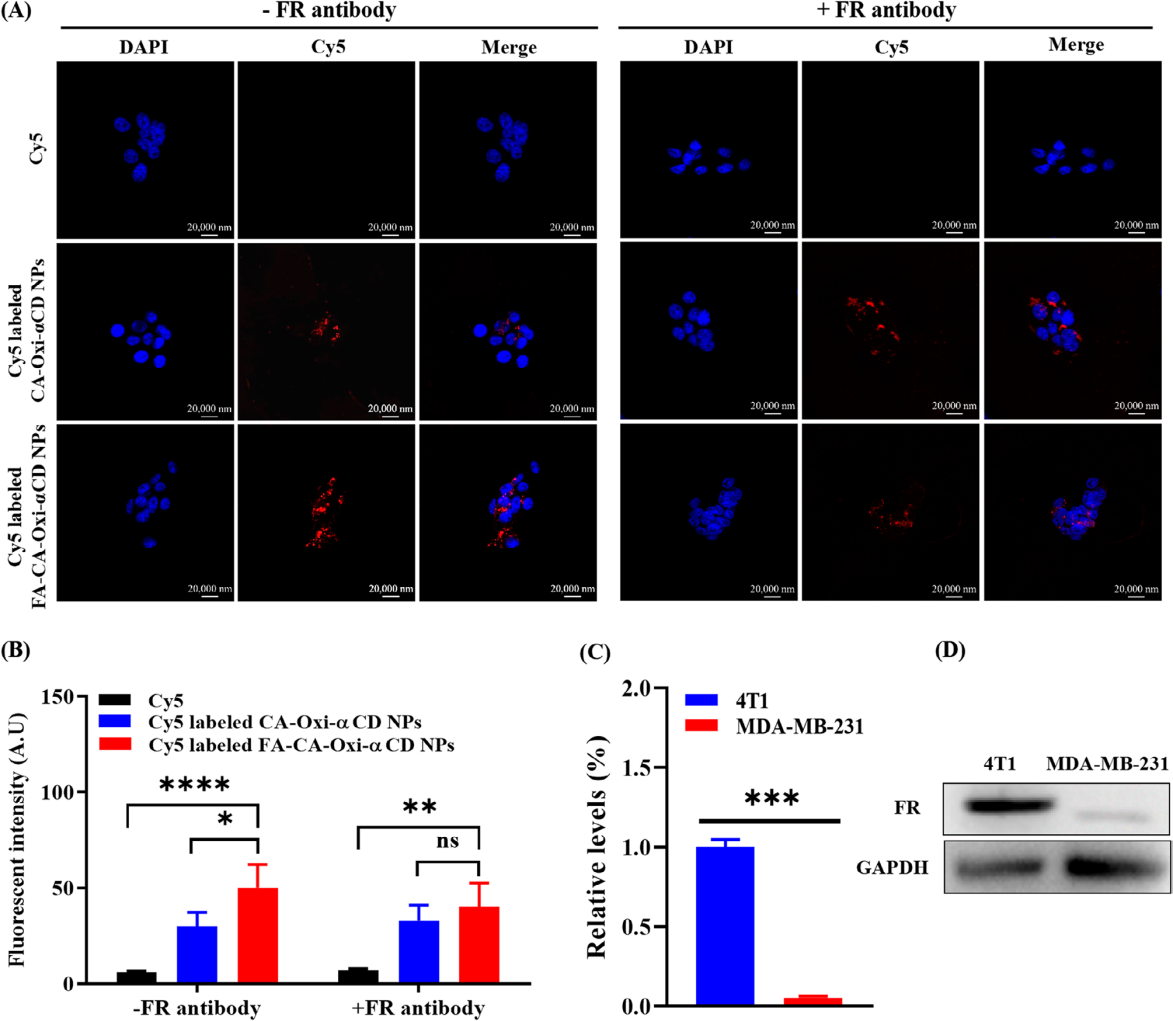
The cellular uptake of 4T1 cells treated with Cy5, Cy5-labeled CA-Oxi-αCD NPs and Cy5-labeled FA-CA-Oxi-αCD NPs for 6 h. (**A**) CLSM images of uptake of 4T1 cells with or without FR pretreatment. DAPI for nuclei staining (blue), Cy5 labeled NPs (red). Scale bar represents 20 μm. (**B**) The semi-quantitative analysis of the corresponding Cy5 fluorescence intensity of intracellular NPs (red) in 4T1 cells with or without FR pretreatment. (**C**) Quantitative analysis of FR expression in 4T1 cells and MDA-MB-231 cells. (**D**) Western blot analysis for the expression of FR in 4T1 cells and MDA-MB-231 cells. *p < 0.05, **p < 0.01, ***p < 0.001, ****p < 0.0001, ns, no significant difference, compared with Cy5-labeled FA-CA-Oxi-αCD NPs (n = 3)

Previous reports have demonstrated that FA modification could improve the targeting capability of NPs to tumor cells with FA receptor overexpression [19]. As shown in Fig. 3A and S4, after 6 h of incubation, the Cy5 fluorescence intensity (red) of the NPs was significantly stronger than that of the free Cy5 group in the 4T1 cells and MDA-MB-231 cells. Meanwhile, compared to the CA-Oxi-αCD NPs group, the fluorescence intensity of Cy5 in the FA-modified CA-Oxi-αCD NPs group was significantly enhanced in the 4T1 cells (Fig. 3A). Fluorescence semiquantitative results also indicated that the FA-modified CA-Oxi-αCD NPs were more easily internalized by 4T1 cells (Fig. 3B). However, the FA modification did not obviously increase the uptake of the CA-Oxi-αCD NPs in MDA-MB-231 cells (Figure S4) because a high expression of FR was detected on the surface of the 4T1 cells but not on the MDA-MB-231 cells (Fig. 3C and D). To further verify FA-mediated endocytosis, FR-blocking experiments were performed. As shown in Fig. 3A and B, after 6 h of incubation, the Cy5 fluorescence intensity in the FA-modified CA-Oxi-αCD NPs with FR antibody-blocked was decreased compared with that in the non-FR antibody-blocked FA-CA-Oxi-αCD NPs and had no significant difference with the CA-Oxi-αCD NPs, suggesting that the FA modification of the NPs significantly facilitated the cellular uptake of the NPs into FA receptor-overexpressed cancer cells.

Whether NPs can penetrate the depth of solid tumors determines their efficacy. Herein, we built 3D tumor spheroids of 4T1 cells to investigate the tumor penetration ability of NPs. As shown in Figure S5, after 6 h of incubation, both the Cy5-labeled CA-Oxi-αCD NPs and the FA-CA-Oxi-αCD NPs penetrated into the deep of tumor spheroids, and the fluorescence intensity was significantly increased compared with that after 2 h of treatment. Similarly, the FA-CA-Oxi-αCD NPs showed the strongest fluorescence intensity compared to the other groups (Figure S5). The results demonstrated that the pH/ROS dual-responsive NPs had excellent penetration into the tumor spheroids, which helps to improve their antitumor efficacy.

### Cell migration and invasion assessment

Tumor cell migration is a prerequisite for invasion and metastasis. Therefore, blocking cell migration is an optional strategy to inhibit tumor invasion and metastasis [41]. Consequently, the effect of NPs on cell migration and invasion of 4T1 cells were investigated. As shown in Figures S6 A and S6 D, the migration of the 4T1 cells was significantly inhibited by DTX or its nanoformulations, but no significant difference was observed between DTX and its nanoformulations group with a 24 h treatment. Importantly, the migration rate of the 4T1 cells was significantly increased in the control, Blank NPs, and DTX groups and slightly increased in the DTX/CA-Oxi-αCD NPs group with a prolonged incubation time, but the migration rate was decreased from 13.98 to 1.71% by the DTX/FA-CA-Oxi-αCD NPs treatment with 48 h of incubation (Figure S6 B and S6 E).

After the treatment with various nanoformulations for 48 h, the invasion of the 4T1 cells was also evaluated. As shown in Figures S6 C and S6 F, compared with the control group and Blank NPs group, cell invasion was slightly inhibited by DTX and the DTX/CA-Oxi-αCD NPs treatment. It is worth noting that the DTX/FA-CA-Oxi-αCD NPs significantly inhibited cell invasion compared with the other treatments (Figure S6 C and S6 F). These results verified that the DTX/FA-CA-Oxi-αCD NPs could significantly inhibit 4T1 cell migration and invasion, which may contribute to the inhibition of cancer metastasis.

### Mitochondria damage and apoptosis

**Fig. 4:**
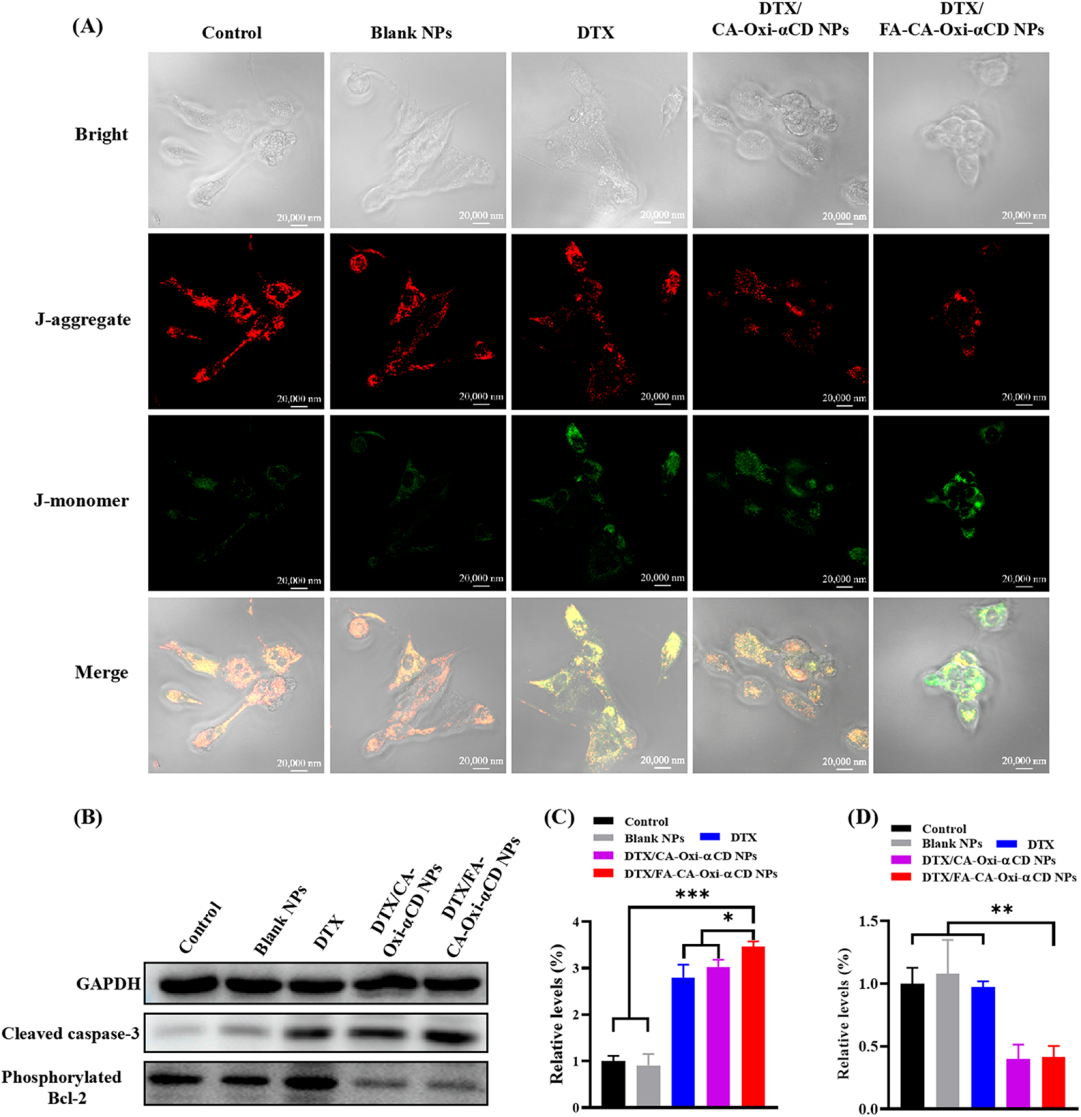
Mitochondrial damage-induced apoptosis of 4T1 cells by control, Blank NPs, DTX, DTX/CA-Oxi-αCD NPs and DTX/FA-CA-Oxi-αCD NPs, respectively. (**A**) CLSM images of JC-1 stained cells after different treatments. Scale bar represents 20 μm. (**B**) Western blot analysis for the expression of cleaved caspase-3 protein, phosphorylated Bcl-2 protein in 4T1 cells with different treatments for 48 h. (**C**) Quantitative analysis of cleaved caspase-3 protein expression in 4T1 cells. (**D**) Quantitative analysis of phosphorylated Bcl-2 protein expression in 4T1 cells. *p < 0.05, **p < 0.01, ***p < 0.001 compared with DTX/FA-CA-Oxi-αCD NPs (n = 3)

Previous investigations demonstrated that CA could induce apoptosis of tumor cells through ROS generation, loss of mitochondrial membrane potential, and caspase activation [42]. However, mitochondria-mediated apoptosis can be inhibited by Bcl-2 anti-apoptotic protein, and it was reported that DTX could trigger Bcl-2 phosphorylation and downregulate the expression of Bcl-2 protein to promote mitochondrial damage and then enhance the antitumor effect [43]. To investigate whether our DTX-loaded NPs could induce mitochondrial damage, we used a JC-1 probe to examine the membrane potential of the 4T1 and MDA-MB-231 cells. The red fluorescence signals transformed into green, suggesting that mitochondria lost their membrane potential and caused significant mitochondrial damage. As shown in Fig. 4A, the green fluorescence signals were weak, and the red fluorescence signals were significant when the cells were treated with cell culture medium or Blank NPs after 48 h of incubation. In contrast, the red fluorescence signals became weak, and the green fluorescence signals (monomers in the cytoplasm) were significant in the 4T1 cells treated with the DTX/CA-Oxi-αCD NPs or the DTX/FA-CA-Oxi-αCD NPs, suggesting that mitochondria lost their membrane potential with NPs treatment. Moreover, the DTX/FA-CA-Oxi-αCD NPs group showed enhanced green fluorescence signals than other group. In the MDA-MB-231 cells, the NPs displayed stronger mitochondrial damage than the free DTX, but there was no significant difference in the targeted and non-targeted groups because low overexpression of FR in the MDA-MB-231 cells (Figure S7, Fig. 3C and D).

To further demonstrate the apoptotic pathway mediated by mitochondria, a Western blot analysis of apoptosis-related proteins was performed. After incubation with the DTX/CA-Oxi-αCD NPs and DTX/FA-CA-Oxi-αCD NPs for 48 h, the expression of caspase-3 protein in 4T1 cells dramatically increased compared with that in the control and Blank NPs groups (Fig. 4B and C). In contrast, the expression of anti-apoptotic phosphorylated Bcl-2 protein was significantly inhibited after the DTX-loaded NPs treatment (Fig. 4B and D). The results demonstrated that pH/ROS dual-responsive NPs decreased the mitochondrial membrane potential and caused severe mitochondrial damage, which in turn led to the generation of more ROS and amplified DTX release, finally induced the tumor cell apoptosis.

### In vitro cytotoxicity of the NPs on tumor cells and intracellular drug release

**Fig. 5:**
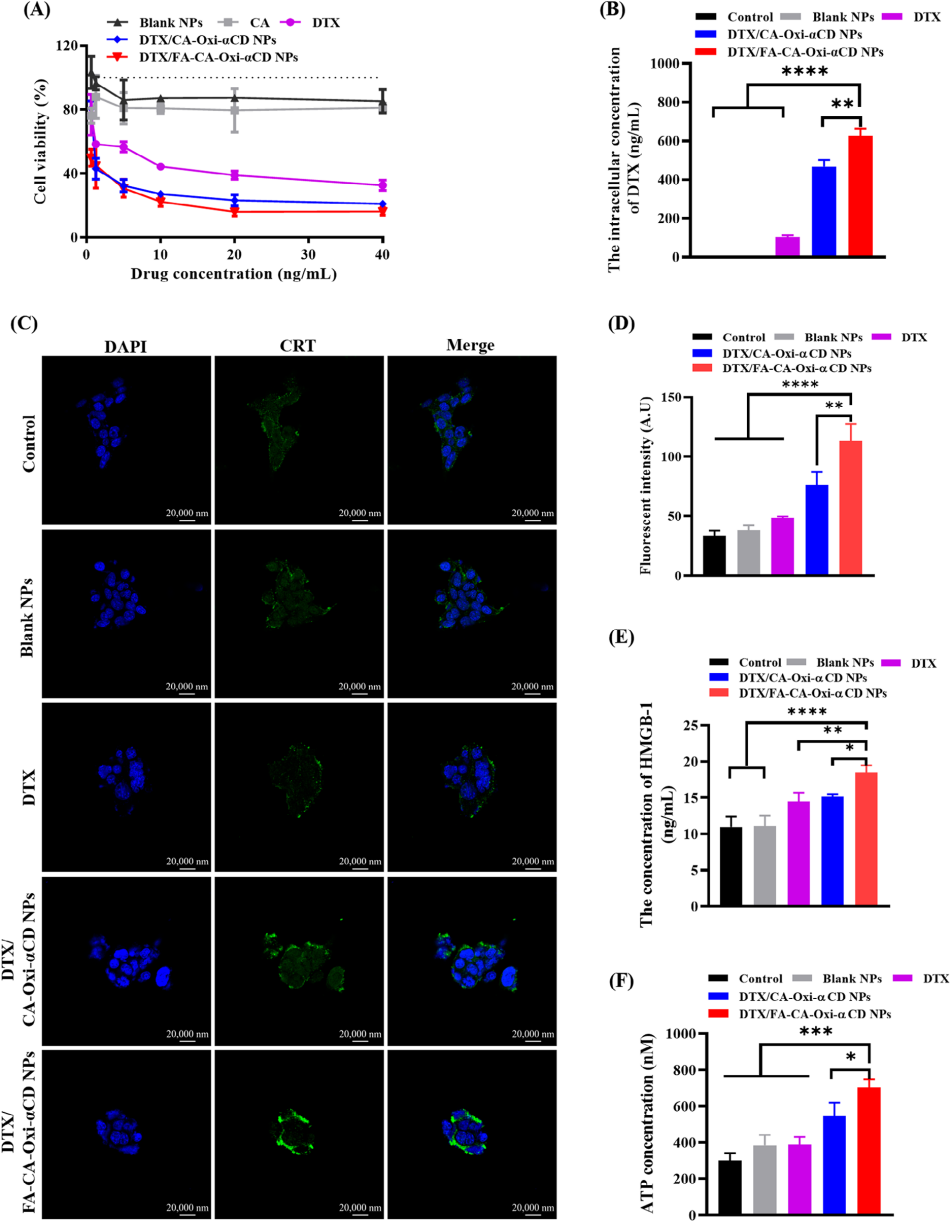
The antitumor effects and ICD induction of NPs on 4T1 cells in vitro. (**A**) Cell viabilities of 4T1 cells after treatment with DTX, CA, Blank NPs, DTX/CA-Oxi-αCD NPs and DTX/FA-CA-Oxi-αCD NPs with various drug concentration for 48 h. (**B**) The intracellular concentration of DTX in 4T1 cells treated with DTX, Blank NPs, DTX/CA-Oxi-αCD NPs and DTX/FA-CA-Oxi-αCD NPs for 48 h. The controls were treated with cell culture medium. (**C**) The CLSM images of CRT expression in 4T1 cells after different treatments. Scale bar represents 20 μm. (**D**) The semi-quantitative analysis of CLSM of the CRT expression in 4T1 cells after different treatments. (**E**) The quantitative determination of HMGB1 release from 4T1 cells after different treatments with ELISA kit. (**F**) ATP secretion from 4T1 cells after different treatments. *p < 0.05, **p < 0.01, ***p < 0.001, and ****p < 0.0001, compared with FA-CA-Oxi-αCD NPs (n = 3)

The cytotoxicity of DTX and the various NPs against 4T1 cells and MDA-MB-231 cells was investigated in vitro. As shown in Fig. 5A and S8 A, both Blank NPs and CA exhibited similar cytotoxicity to the 4T1 cells and MDA-MB-231 cells due to the released CA from the Blank NPs or the free CA having certain antitumor activity [44]. Compared with the DTX group, the DTX/CA-Oxi-αCD NPs and DTX/FA-CA-Oxi-αCD NPs significantly decreased the viability of the 4T1 cells with increasing drug concentrations (Fig. 5A). Furthermore, the half-maximal inhibitory concentration (IC_50_) of nanoformulations on the 4T1 and MDA-MB-231 cells was calculated and listed in Table S2. The IC_50_ of DTX was 3.5-fold or 9.8-fold higher than that of the DTX/CA-Oxi-αCD NPs or the DTX/FA-CA-Oxi-αCD NPs, indicating that the DTX/FA-CA-Oxi-αCD NPs showed enhanced cytotoxicity to the 4T1 cells. For the MDA-MB-231 cells, the DTX/FA-CA-Oxi-αCD NPs also exhibited the strongest cell inhibitory activity compared with the DTX and DTX/CA-Oxi-αCD NPs, which validated that FA modification increased the antitumor activity of DTX compared with the unmodified NPs. The results were further confirmed by the intracellular drug release of NPs. As shown in Fig. 5B, the intracellular DTX concentration in the DTX/FA-CA-Oxi-αCD NPs was significantly higher than that in the non-targeted group in 4T1 cells, indicating that FA modification enhanced the endocytosis of the NPs by 4T1 cells. For the MDA-MB-231 cells, the intracellular DTX concentration in the DTX/FA-CA-Oxi-αCD NPs was similar to that in the DTX/CA-Oxi-αCD NPs (Figure S8 B), and both were significantly higher than that in the free DTX group (Fig. 5B and S8 B).

### NPs induced immune activation in vitro

We also investigated the effect of DTX and various NPs on immune activation in vitro. CRT expressed on the surface of immunogenic dead cells acts as an “eat me” signal to induce antigen presentation and infiltration of tumor-specific cytotoxic T lymphocytes, effectively activating immune responses [37]. Figure 5 C shows that DTX/CA-Oxi-αCD NPs and DTX/FA-CA-Oxi-αCD NPs caused a higher levels of CRT exposure on 4T1 tumor cell surface than the control, Blank NPs, and DTX, especially DTX/FA-CA-Oxi-αCD NPs, which induced significant exposure of CRT on the surface of the 4T1 cells compared to the DTX/CA-Oxi-αCD NPs. Moreover, the semi-quantitative analysis of CLSM images showed that the mean fluorescence intensity of CRT in the 4T1 cells treated with the DTX/FA-CA-Oxi-αCD NPs was obviously enhanced compared to the other treatment groups (Fig. 5D).

HMGB1 can bind to Toll-like receptor 4 to promote the antigen-presenting ability of dendritic cells [45]. Autophagy promotes the release of ATP from dying cells, constituting a “find-me” signal and proinflammatory stimulus for the recruitment of dendritic cells and their precursors [46]. Figure 5E shows that DTX/FA-CA-Oxi-αCD NPs significantly increased HMGB1 release compared with the other treatment groups. It is worth noting that the DTX/FA-CA-Oxi-αCD NPs caused the highest levels of ATP secretion among all the treatment groups (Fig. 5F).

Taken together, the DTX/FA-CA-Oxi-αCD NPs caused the highest levels of CRT exposure, HMGB-1 release, and ATP secretion among all the treatment groups. These results suggested that the DTX/FA-CA-Oxi-αCD NPs could induce the highest level of ICD in vitro, which would further promote tumor-specific immune responses.

### In vivo biodistribution

**Fig. 6:**
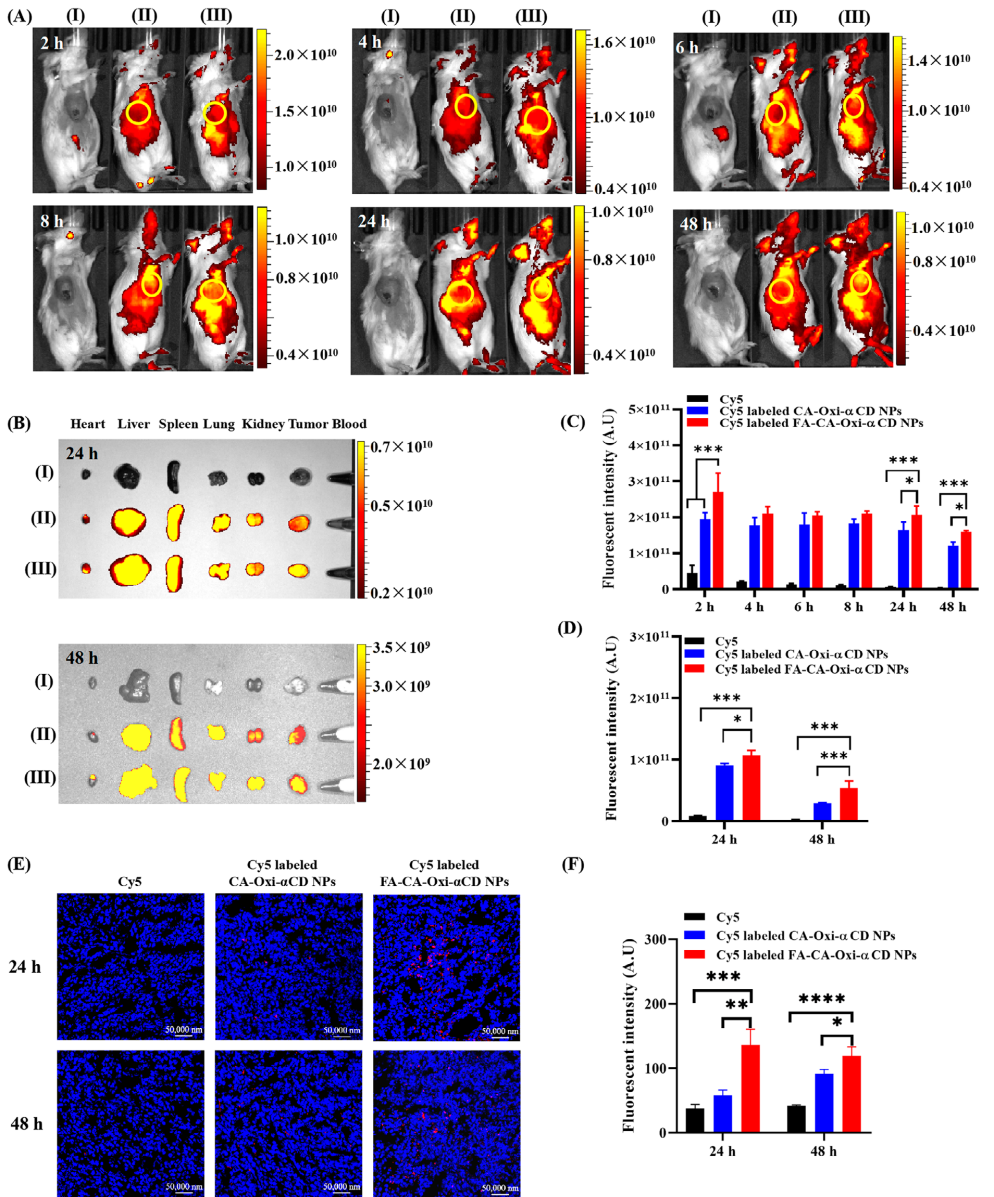
In vivo fluorescence distribution of 4T1 tumor-bearing mice after intravenous administration of free Cy5, Cy5-labeled CA-Oxi-αCD NPs and Cy5-labeled FA-CA-Oxi-αCD NPs. (**A**) In vivo images of mice after 2 h, 4 h, 6 h, 8 h, 24 and 48 h injection. (**B**) Ex vivo fluorescence image of the excised tumors and major tissues at 24 and 48 h post-injection. (**C**) Semi-quantitative analysis of fluorescence intensity of mice after 2 h, 4 h, 6 h, 8 h, 24 and 48 h injection in vivo. (**D**) Semi-quantitative analysis of fluorescence intensity in excised tumor at 24 and 48 h. (**E**) CLSM images of tumor sections from mice after different treatments. Red, Cy5-labeled NPs, blue, cell nuclei stained with DAPI. Scale bar represents 50 μm. (**F**) Semi-quantitative analysis of CLSM images of tumor tissues from mice with different treatment at 24 and 48 h. (I) free Cy5, (II) Cy5-labeled CA-Oxi-αCD NPs, (III) Cy5-labeled FA-CA-Oxi-αCD NPs. *p < 0.05, **p < 0.01, ***p < 0.001, ****p < 0.0001, compared with Cy5-labeled FA-CA-Oxi-αCD NPs (n = 3)

The distribution and accumulation of the pH/ROS dual-responsive NPs in vivo were evaluated in the 4T1 tumor-bearing mice after intravenous administration. As shown in Fig. 6A, the fluorescence signals of Cy5 were quickly attenuated in the free Cy5 group, indicating that Cy5 was rapidly eliminated in the body. In contrast, the Cy5-labeled CA-Oxi-αCD NPs and the FA-CA-Oxi-αCD NPs mainly accumulated in the tumor site and maintained stronger fluorescence signals after 48 h of injection (Fig. 6A and C). Compared to the Cy5/CA-Oxi-αCD NPs group, the Cy5/FA-CA-Oxi-αCD NPs exhibited stronger fluorescence signals at the tumor site after 24 and 48 h of administration (Fig. 6A and C) due to FA-mediated targeting. The ex vivo images also demonstrated that FA modification significantly improved the tumor targeting capacity of the NPs compared to their non-targeted counterparts after 24 and 48 h of administration (Fig. 6B). Semi-quantitative analysis of total radiant efficiency showed that the Cy5-labeled FA-CA-Oxi-αCD NPs had 1.85-fold and 24.06-fold increases in fluorescence intensity at the tumor site compared to the CA-Oxi-αCD NPs and the free Cy5 group 48 h post-injection, respectively (Fig. 6D). The result was further confirmed by the images of the frozen section of the tumor tissue. As shown in Fig. 6E and F, the FA-CA-Oxi-αCD NPs had higher tumor accumulation and penetration than the CA-Oxi-αCD NPs and the free Cy5. These results demonstrated that the Cy5/CA-Oxi-αCD NPs, especially the Cy5/FA-CA-Oxi-αCD NPs, effectively accumulated at the tumor region and penetrated into the interior of the tumor, which is beneficial for delivering therapeutics into tumors.

### In vivo antitumor efficacy, antimetastatic activity, and survival curves

**Fig. 7:**
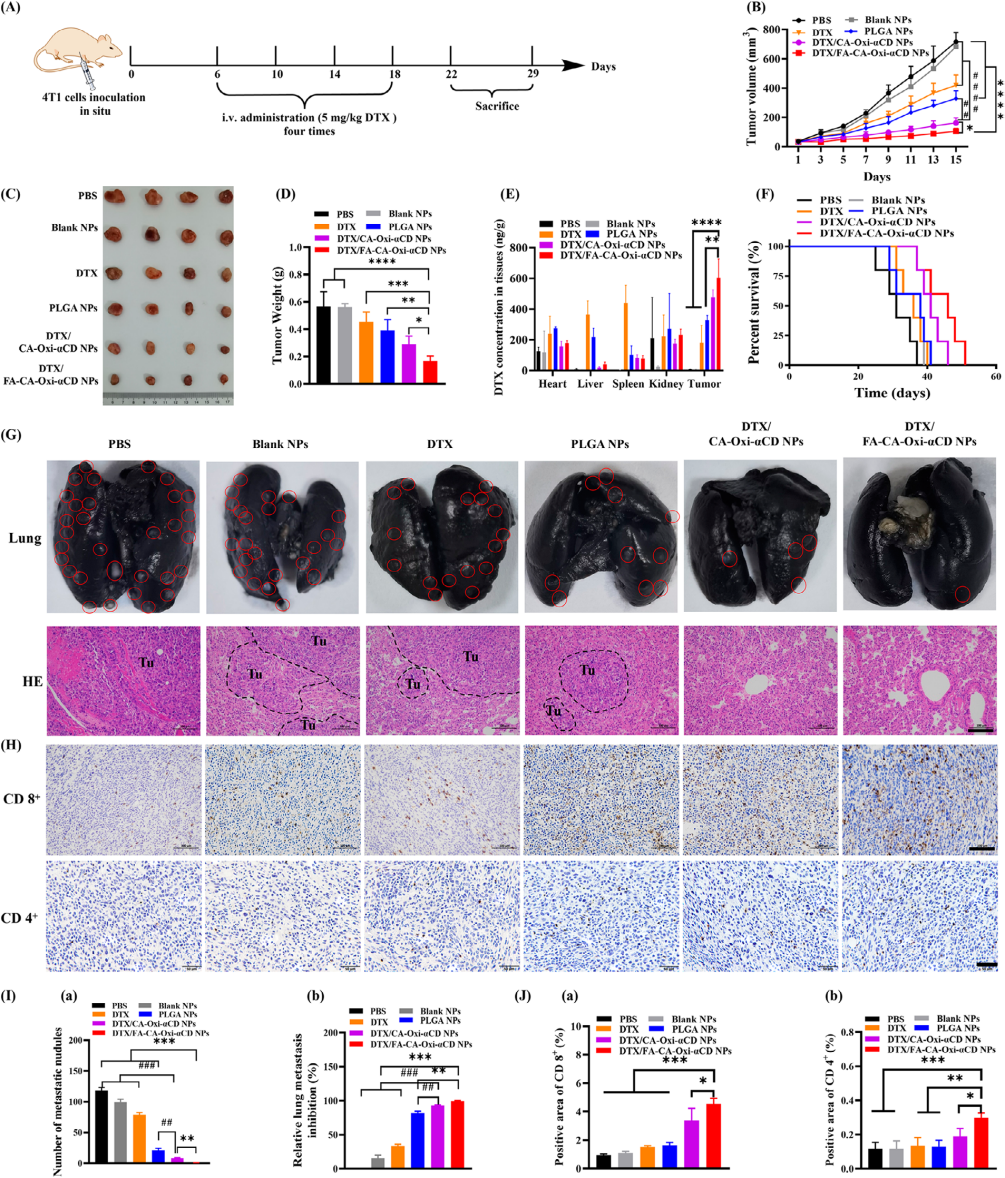
In vivo antitumor and antimetastasis efficacy evaluation. (**A**) Scheme illustration of the in vivo antitumor experiment design. (**B**) Tumor growth curves after intravenous injection of free DTX and various NPs. (**C**) Representative photographs of tumor tissue from mice in different groups. (**D**) The tumor weight of mice in different groups. (**E**) The DTX concentration in major tissues of different groups. (**F**) The survival rate of mice after various treatment. (**G**) Representative images of lung tissues harvested from mice in different groups and histological examination of the pulmonary metastatic foci in different groups by H&E staining. Scale bar represents 100 μm. (**H**) Immunohistochemistry assay for CD 8^+^ (upper panel) and CD 4^+^ ((lower panel) in tumor tissues. Scale bar represents 100 μm or 50 μm, respectively. (**I**) (a) The number of visually detected metastatic nodules in the lungs of different groups, (b) The inhibition rate on lung metastasis of different groups. (**J**) (a) The semi-quantitative analysis of the CD8^+^ expression and (b) CD4^+^ expression in tumor tissues from mice after different treatments. *p < 0.05, **p < 0.01, ***p < 0.001 and ****p < 0.0001, compared with DTX/FA-CA-Oxi-αCD NPs. ^#^*p* < 0.05, ^##^*p* < 0.01, ^###^*p* < 0.001 and ^####^*p* < 0.0001, compared with DTX/CA-Oxi-αCD NPs

The in vitro experiments of the pH/ROS dual-responsive NPs inspired us to investigate their in vivo therapeutic efficacies. The antitumor efficacy of various NPs was investigated in vivo in a 4T1 tumor-bearing mouse model. As shown in Figures S9 A and S9 B, all mice maintained their body weight and had no detected hepatic or kidney toxicity. Compared to the PBS or Blank NPs group, tumor growth was significantly suppressed by the DTX treatment (Fig. 7B and C). The DTX/PLGA NPs exhibited enhanced antitumor activity compared to DTX (Fig. 7B and C). And tumor growth was remarkably inhibited by the DTX/CA-Oxi-αCD NPs compared to the DTX/PLGA NPs (Fig. 7B and C), indicating that the pH/ROS dual responsive NPs exhibit excellent antitumor activity than the non-responsive NPs. Importantly, the DTX/FA-CA-Oxi-αCD NPs resulted in the greatest tumor suppression among all treatment groups (Fig. 7B and C), which further validated that FA modification could enhance the targeting capability of NPs to tumors. The tumor weight further confirmed that the FA modification increased the antitumor efficacies of the NPs (Fig. 7D). We also determined the DTX contents in the tumor site by LC/MS/MS. The DTX/FA-CA-Oxi-αCD NPs had the highest drug concentrations in tumors than in other tissues due to FA-mediated targeting, and the DTX accumulation in tumor in DTX/FA-CA-Oxi-αCD NPs group was significantly more than in the DTX and PLGA NPs groups, suggesting that the improved antitumor efficacy of DTX/FA-CA-Oxi-αCD NPs compared to the other groups was mainly attributed to the release of more DTX at the tumor sites (Fig. 7E and S9 C). To further investigate the antitumor efficacies of the pH/ROS dual-responsive NPs, the survival span of the tumor-bearing mice was recorded with the various therapeutic treatments. As shown in Fig. 7F, the FA modified DTX/CA-Oxi-αCD NPs extended the survival span of the mice compared to the non-targeted group.

We also investigated the inhibition of spontaneous pulmonary metastasis in the 4T1 orthotopic mammary tumor model by the NPs. The lung tissues of the tumor-bearing mice exhibited numerous metastatic foci in the PBS and Blank NPs groups after 29 days of treatment (Fig. 7G). DTX treatment relatively attenuated the number of white deposits in the lung (Fig. 7G). It is worth noting that all the DTX-loaded NPs significantly reduced metastatic foci in the lung (Fig. 7G). In particular, the FA-modified pH/ROS dual-responsive NPs displayed the best inhibition of metastasis compared to the other treatment groups (Fig. 7G). Quantitative analysis of tumor nodules also demonstrated that the DTX/CA-Oxi-αCD NPs significantly blocked metastasis compared to other groups (Fig. 7I (a)). Importantly, only a few metastatic nodules were counted in the lung tissues with the DTX/FA-CA-Oxi-αCD NPs treatment, which reached 99.72 ± 0.49% inhibition of lung metastases (Fig. 7I (b)). In addition, H&E staining of the lung also validated that the pH/ROS dual-responsive NPs displayed enhanced tumor pulmonary metastasis suppression (Fig. 7G).

Terminal deoxynucleotidyl transferase (TdT)-mediated dUTP nick end labeling (TUNEL) immunofluorescence staining results showed that much larger areas of tumor apoptosis (green color) were observed in the DTX/CA-Oxi-αCD NPs group than in the DTX and DTX/PLGA NPs groups (Figure S10 A), and tumor apoptosis was further increased with the FA-modified NPs treatment (Figure S10 A). Immunohistochemistry staining assays were also conducted to further confirm the enhanced antitumor activity of the DTX-loaded pH/ROS dual-responsive NPs based on cell apoptosis. As shown in Figure S10 B, the DTX/FA-CA-Oxi-αCD NPs significantly decreased Ki67 expression compared to other groups, demonstrating that the proliferation of tumor cells was significantly inhibited. Meanwhile, the DTX/CA-Oxi-αCD NPs induced severe cell apoptosis with high expression of caspase-3, and the caspase-3 expression was further increased with targeted NPs treatment, which was consistent with the TUNEL results in the tumor tissues (Figure S10 C). TUNEL analysis and immunohistochemistry staining results revealed that the DTX-loaded pH/ROS dual-responsive NPs dramatically blocked tumor growth by inhibiting cell proliferation and inducing tumor cell apoptosis.

An enlarged spleen can sometimes be observed in cancer patients treated with chemotherapy, indicating the negative regulation of the immune system. Reduction in the spleen size may imply a systemic immunomodulating effect induced by the NPs [47]. Our in vivo efficacy experiments revealed that the spleen weight of the untreated 4T1 tumor-bearing mice was approximately 1.5 times that of the DTX and DTX/PLGA NPs groups and 1.9 times and 2.7 times that of the DTX/CA-Oxi-αCD NPs and DTX/FA-CA-Oxi-αCD NPs, respectively (Figure S11). In tumor-bearing organisms, the function of CD8^+^ T cells is usually inhibited or even undergoes apoptosis due to the immunosuppressive effect of the tumor, thus enabling tumors to acquire the ability of immune escape. Accordingly, we analyzed the expression of CD8^+^ and CD4^+^ in tumor tissues of the mice by immunohistochemistry (Fig. 7H). The quantitative analysis in Fig. 7J shows that the expression of CD8^+^ and CD4^+^ in the tumor tissues from the mice treated with DTX/CA-Oxi-αCD NPs and DTX/FA-CA-Oxi-αCD NPs were greatly upregulated compared with that treated with DTX and DTX/PLGA NPs, suggesting that the DTX-loaded pH/ROS dual-responsive NPs effectively alleviated splenomegaly and the immunosuppressive state of tumor.

Taken together, the in vivo results confirmed that the DTX/CA-Oxi-αCD NPs, especially the DTX/FA-CA-Oxi-αCD NPs, enhanced antitumor efficacies and antimetastatic effects compared with other treatments by inducing tumor cell apoptosis and relieving the immunosuppressive state in the tumor immune microenvironment.

### Pulmonary metastasis inhibition

**Fig. 8:**
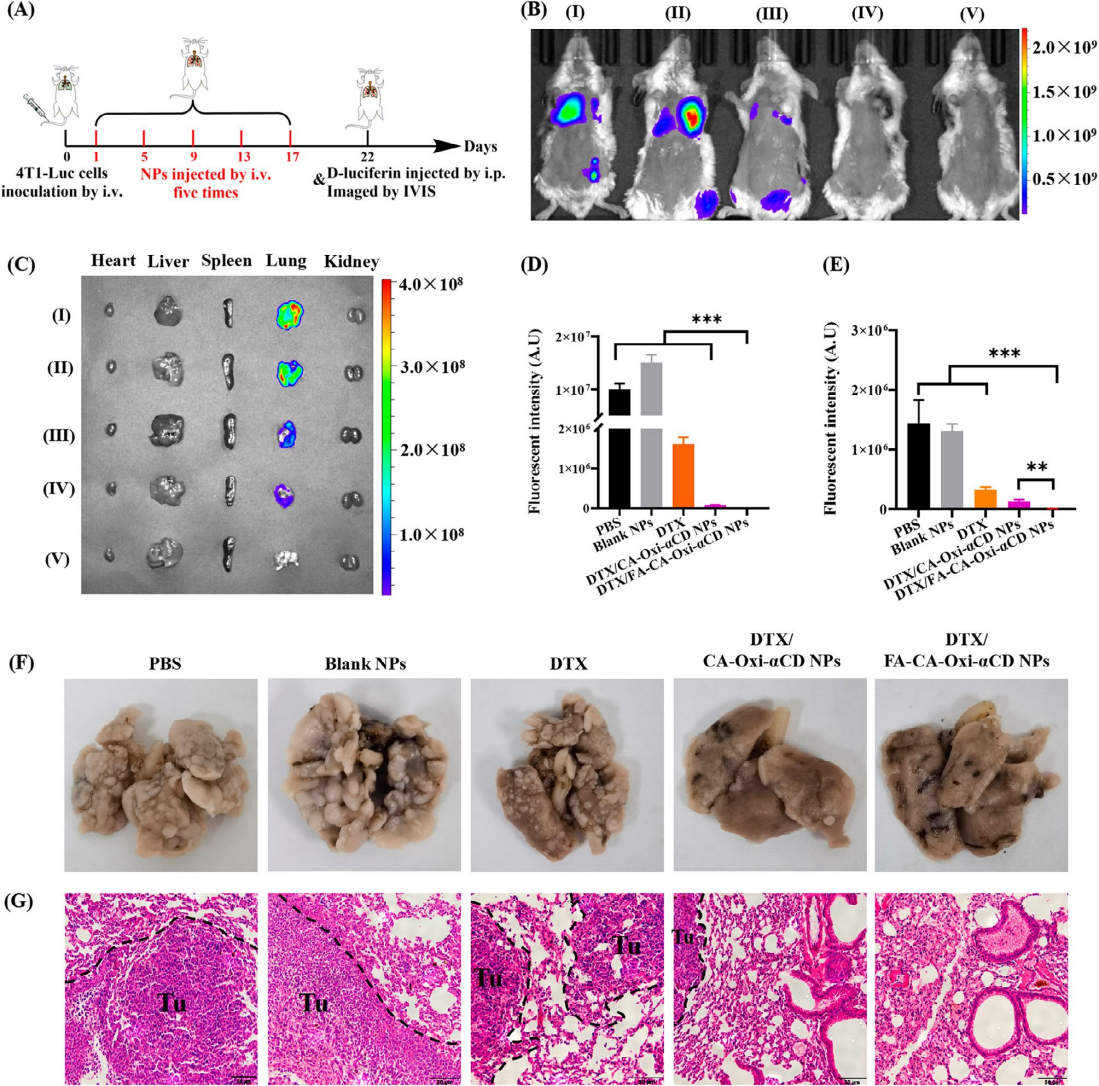
In vivo bioluminescent distribution of the mice treated with PBS, Blank NPs, DTX, DTX/CA-Oxi-αCD NPs and DTX/FA-CA-Oxi-αCD NPs at the same doses of 5 mg/kg of DTX (n = 5). (**A**) Scheme illustration of the pulmonary metastasis inhibition experiment design. (**B**) In vivo bioluminescent images of the mice treated with DTX and various NPs. (**C**) The bioluminescence images of the ex vivo major organs from mice after different treatments. (**D**) Bioluminescence intensity in the lung of mice after different treatments in vivo. (**E**) Bioluminescence intensity of the ex vivo lungs in the mice after different treatments. (**F**) The photo images of ex vivo lungs in the mice treated with DTX and various NPs. (**G**) Representative microphotographs of H&E sections of lungs in the mice treated with DTX and various NPs. Scale bar represents 50 μm. (I) PBS, (II) Blank NPs, (III) DTX, (IV) DTX/CA-Oxi-αCD NPs, (V) DTX/FA-CA-Oxi-αCD NPs. *p < 0.05, **p < 0.01, and ***p < 0.001, compared with DTX/ FA-CA-Oxi-αCD NPs (n = 5)

Previous results have confirmed that DTX/CA-Oxi-αCD NPs can inhibit the migration and invasion of 4T1 cell in vitro and lung metastasis in vivo. To further investigate the antimetastasis effect of the DTX-loaded pH/ROS dual-responsive NPs, we established a lung metastasis model via tail vein injection of 4T1 cells in BALB/c mice. At 22 days after injection of 4T1-luc cells, bioluminescent images of mice were obtained with an IVIS Spectrum Imaging System. As shown in Fig. 8B and D, remarkable luminescence intensity was found in the lungs of the mice treated with PBS and Blank NPs, and the luminescence signals in the lungs of mice were significantly decreased with DTX treatment. Furthermore, weak luminescence signals were found in the DTX/CA-Oxi-αCD NPs group (Fig. 8B), demonstrating that metastasis was further inhibited with the pH/ROS dual-responsive NPs treatment. Importantly, almost no bioluminescence signals were observed in the mice treated with the DTX/FA-CA-Oxi-αCD NPs (Fig. 8B), implying that metastasis was thoroughly inhibited by the DTX/FA-CA-Oxi-αCD NPs. In addition, quantitative analysis results also demonstrated that the 4T1-luc cells lung metastasis could be significantly inhibited by the DTX-loaded pH/ROS dual-responsive NPs (Fig. 8D). The result was further confirmed by bioluminescent imaging of the ex vivo lung tissues. As shown in Fig. 8C, the luminescence intensity of the ex vivo lung was attenuated with the DTX treatment and further decreased by the DTX/CA-Oxi-αCD NPs treatment (Fig. 8C). Interestingly, almost no luminescence signals were observed in excised pulmonary tissues of the DTX/FA-CA-Oxi-αCD NPs group (Fig. 8C), which is in accordance with the in vivo imaging results. Notably, the luminescence intensity in the ex vivo lung treated with the DTX/FA-CA-Oxi-αCD NPs was approximately 20 times lower than that of the DTX/CA-Oxi-αCD NPs (Fig. 8E), confirming that FA modification significantly blocked tumor metastasis. In addition, the photo images and H&E staining images also confirmed that the DTX/FA-CA-Oxi-αCD NPs remarkably blocked tumor cell metastasis to the lung tissues (Fig. 8F and G).

### Chemotherapy and immunotherapy combination treatment

**Fig. 9:**
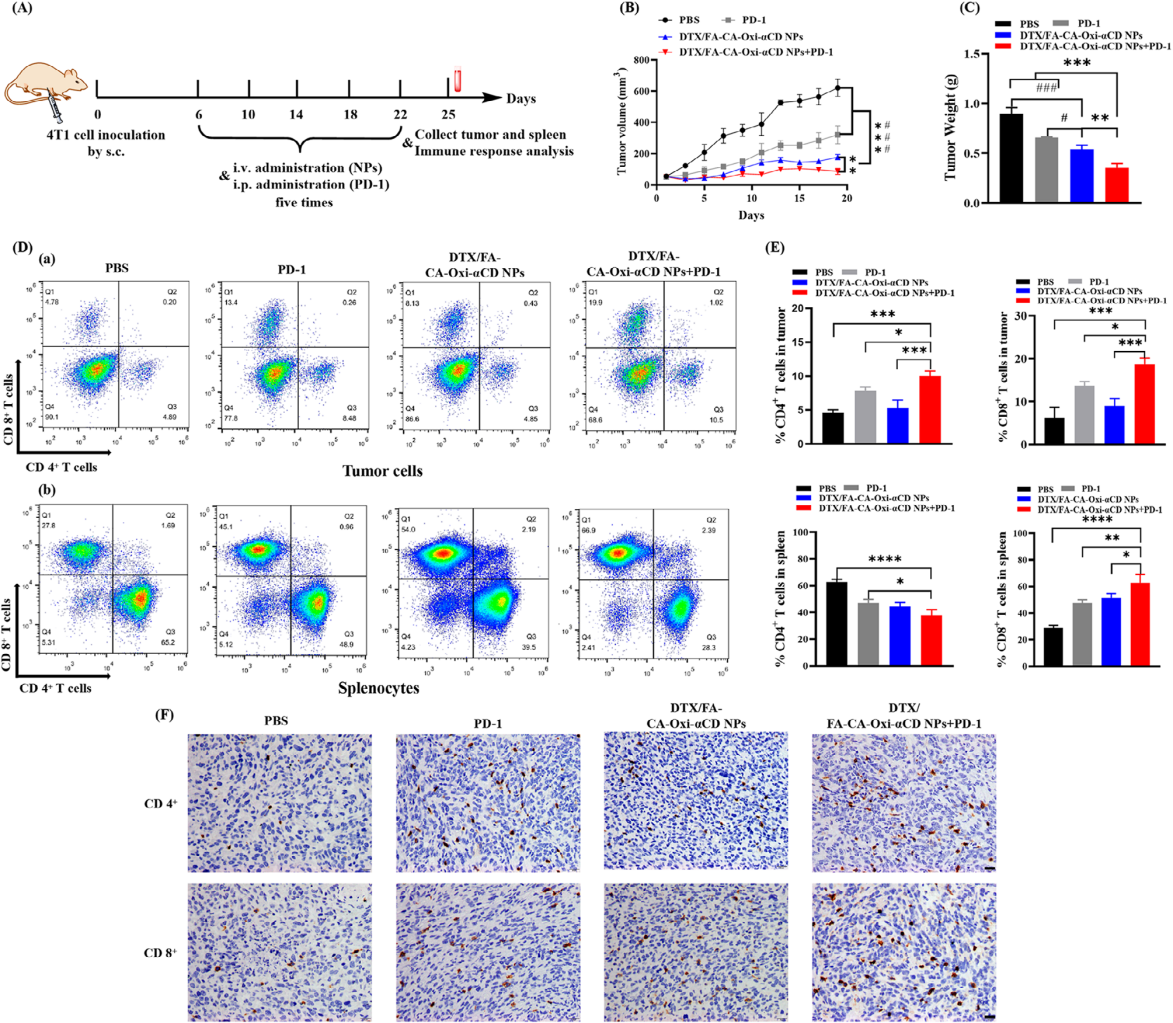
The antitumor ability of chemotherapy and immunotherapy combination therapy and the ability to stimulate antitumor immune responses in vivo. (**A**) Scheme illustration of the chemotherapy and immunotherapy combination treatment. (**B**) Tumor growth curves of mice after different treatments. (**C**) The tumor weight of mice in different treatment groups. (**D**) (a) Representative flow cytometric plots of CD8^+^ T cells and CD4^+^ T cells from mouse tumors after different treatments. (b) Representative flow cytometric plots of CD8^+^ T cells and CD4^+^ T cells from mouse splenocytes after different treatments. (**E**) Proportions of CD8^+^ T cells and CD4^+^ T cells from tumors or splenocytes were determined by flow cytometry after different treatments, respectively. (**F**) The immunohistochemical staining of CD8^+^ T and CD4^+^ T cells in mice tumors after different treatments. Brown regions indicate the presence of CD8^+^ T cells or CD4^+^ T cells. Scale bar represents 20 μm. *p < 0.05, **p < 0.01, ***p < 0.001 and ****p < 0.0001, compared with DTX/FA-CA-Oxi-αCD NPs + anti-PD-1 antibody. ^#^*p* < 0.05, ^##^*p* < 0.01, ^###^*p* < 0.001, compared with DTX/FA-CA-Oxi-αCD NPs

As mentioned above, DTX-loaded pH/ROS dual-responsive NPs can induce the highest level of ICD in vitro and activate CD8^+^ T cells in vivo, which may increase the immunotherapy efficacy of tumors. Consequently, we hypothesized that DTX-loaded pH/ROS dual-responsive NPs combined with anti-PD-1 antibody may increase the antitumor therapeutic effect of anti-PD-1 antibody. To verify this hypothesis, we evaluated the combined therapeutic effect of DTX/FA-CA-Oxi-αCD NPs and anti-PD-1 antibodies in 4T1 tumor-bearing mice. All mice had no obvious changes in body weight during treatment (Figure S12 A). As shown in Fig. 9B and S12 B, the DTX/FA-CA-Oxi-αCD NPs with anti-PD-1 antibody combination treatment significantly inhibited tumor growth compared with other groups. The size and weight of the tumors separated from the sacrificed mice treated with the DTX/FA-CA-Oxi-αCD NPs combined with anti-PD-1 antibody exhibited optimal therapeutic efficacy compared to other groups (Fig. 9B and C). The above results indicated that the DTX/FA-CA-Oxi-αCD NPs enhanced immunotherapy of PD-1 on TNBC. The CD8^+^ T cells and CD4^+^ T cells were detected by flow cytometry and immunohistochemical staining. As shown in Fig. 9D (a) and 9E, the DTX/FA-CA-Oxi-αCD NPs with PD-1 group had higher percentages of the two types of T cells in the tumors than the PD-1 group. Immunohistochemistry also demonstrated more extensive brown areas in the tumor tissues found in the DTX/FA-CA-Oxi-αCD NPs combined with anti-PD-1 antibody group, indicating the higher expression of CD8^+^ and CD4^+^ in the tumor tissues (Fig. 9F).

We also investigated immune responses in the spleen. The DTX/FA-CA-Oxi-αCD NPs combined with anti-PD-1 antibody treatment significantly reduced splenomegaly compared with the other treatments (Figure S13). This combined treatment caused 1.33-fold CD8^+^ T cells in the spleens compared to the PD-1 group (Fig. 9D (b) and 9E). However, there were fewer CD4^+^ T cells in the spleen from the mice treated with DTX/FA-CA-Oxi-αCD NPs combined with anti-PD-1 antibody than in the PD-1 group (Fig. 9D (b) and 9E). These results suggest that CD8^+^ T cells may be the main effector cells of the antitumor responses in the TNBC model. Overall, the pH/ROS dual-responsive NPs efficiently killed the tumor cells, produced tumor-associated antigens, and further activated T cells, which may explain why the combination therapy so effectively inhibited tumor growth and improved the antitumor efficacy of anti-PD-1 antibody.

### In vivo safety evaluation

As shown in Figure S14, compared to saline, the NPs did not affect the blood chemistry of the mice, including the number of white blood cells (WBCs), red blood cells (RBCs), and platelets (PLTs), as well as the level of hemoglobin (HGB). There were no significant differences between the saline and different dosages of the NPs. Furthermore, alanine transaminase (ALT), aspartate transaminase (AST), creatinine (CR), and urea (UREA) related to the functions of the liver and kidney of the mice verified that no obvious hepatic and kidney toxicity was observed after the NPs treatments. In addition, the corresponding histological changes in organs were analyzed by H&E staining. No obvious organ damage could be observed among all the groups, suggesting that the designed and prepared NPs at high doses were excellently tolerable and had no significant acute side effects on the mice (Figure S15).

## Conclusions

In this study, we developed pH/ROS-responsive NPs to deliver the ICD inducer DTX to enhance immunotherapy and inhibit lung metastasis. These pH/ROS-responsive NPs can self-supply ROS to accelerate payload release in the TME. In vitro experiments validated that the DTX-loaded pH/ROS-responsive NPs were effectively internalized by FA-overexpressed cells and penetrated deep into tumor spheroids. Meanwhile, the DTX-loaded pH/ROS-responsive NPs significantly blocked 4T1 cell migration and decreased cell invasion, which may be advantageous for inhibiting the metastasis of TNBC. In vivo experiments demonstrated that the DTX/FA-CA-Oxi-αCD NPs effectively accumulated at the tumor site, and the accumulated NPs remarkably blocked tumor growth and inhibited tumor metastasis. Moreover, the pH/ROS dual-responsive NPs significantly improved immune responses in vivo. The reason may be that the DTX/FA-CA-Oxi-αCD NPs promoted the ICD effect, triggered effector T-cell activation, and relieved the immunosuppressive state of the TME. In summary, the DTX-loaded pH/ROS dual-responsive NPs achieved encouraging therapeutic efficacy and demonstrated great potential in preventing tumor metastasis, inhibiting tumor growth, and improving the efficacy of anti-PD-1 antibody.

### Electronic supplementary material

Below is the link to the electronic supplementary material.

Supplementary Material 1

## Data Availability

The datasets used and/or analysed during the current study are available from the corresponding author on reasonable request.

## List of Abbreviations

ICD: Immunogenic cell death
TME: Tumor microenvironment
ROS: Reactive oxygen species
CA: Cinnamaldehyde
DTX: Docetaxel
TNBC: Triple-negative breast cancer
PD-L1: Programmed death protein ligand 1
CRT: Calreticulin
HMGB1: High mobility group Box 1
ATP: Adenosine-5′-triphosphate
DDS: Drug delivery system
HPAP: 4-hydroxymethylphenylboronic acid pinacol ester
CD: Cyclodextrin
FA: Folic acid
FR: Folate receptor
DMEM: Dulbecco’s modified Eagle’s medium
DLS: Dynamic light scattering
HPLC: High-performance liquid chromatography
DL: Drug loading
EE: Encapsulation efficiency
CLSM: Confocal laser scanning microscopy
PDI: Polymer dispersity index
TEM: Transmission electron microscopy
TUNEL: Terminal deoxynucleotidyl transferase (TdT)-mediated dUTP nick end labeling
WBCs: White blood cells
RBCs: Red blood cells
PLTs: Platelets
HGB: Hemoglobin
ALT: Alanine transaminase
AST: Aspartate transaminase
CR: Creatinine
UREA: Urea
H&E staining: Hematoxylin-eosin staining
