# Supplementary material for: Docetaxel-loaded pH/ROS dual-responsive nanoparticles with self-supplied ROS for inhibiting metastasis and enhancing immunotherapy of breast cancer
Source: J Nanobiotechnology. 2023 Aug 22;21:286. doi: 10.1186/s12951-023-02013-y (PMC10464340; doi:10.1186/s12951-023-02013-y)
Supplement: Supplementary file 1 — Supplementary Material 1 [file 12951_2023_2013_MOESM1_ESM.docx]

### Supporting Information

**Docetaxel-loaded pH/ROS dual-responsive nanoparticles with self-supplied ROS for inhibiting metastasis and enhancing immunotherapy of breast cancer**

Yu Wang ^1,†^, Qianmei Wang^1,2,†^, Xiaowen Wang^1,†^, Pu Yao^1^, Qing Dai^1^, Xiaowei Qi^3^, Ming Yang^4^, Xiao Zhang^5^, Rong Huang^2^, Jing Yang^2^, Qian Wang^1^, Peiyuan Xia^1,*^, Dinglin Zhang^2,6,*^, Fengjun Sun^1,*^

1 Department of Pharmacy, Southwest Hospital, Army Medical University (Third Military Medical University), Chongqing 400038, China

2 Department of Chemistry, College of Basic Medicine, Army Medical University (Third Military Medical University), Chongqing 400038, China

3 Department of Breast and Thyroid Surgery, Southwest Hospital, Army Medical University (Third Military Medical University), Chongqing 400038, China

4 Department of Pharmacy, Affiliated Hospital of North Sichuan Medical College, Nanchong 637000, China

5 Department of Stem Cell and Regenerative Medicine, Southwest Hospital, Army Medical University (Third Military Medical University), Chongqing 400038, China

6 Department of Urology, Southwest Hospital, Third Military Medical University (Amy Medical University), Chongqing 400038, China

*Corresponding authors:

Fengjun Sun, PhD, Associate Prof.

Department of Pharmacy,

Southwest Hospital,

Army Medical University (Third Military Medical University), Chongqing 400038, China

1. mail: [fengj_sun@163.com](mailto:fengj_sun@163.com,)

ORCID: 0000-0003-2679-8797

Dinglin Zhang, PhD, Prof.

Department of Chemistry,

College of Basic Medicine,

Army Medical University (Third Military Medical University), Chongqing 400038, China

Email: zh18108@163.com, [zh18108@tmmu.edu.cn](mailto:zh18108@tmmu.edu.cn)

ORCID: 0000-0003-4400-919X

Peiyuan Xia, PhD, Prof.

Department of Pharmacy,

Southwest Hospital,

Army Medical University (Third Military Medical University), Chongqing 400038, China

E-mail: [peiyuan_xia2013@163.com](mailto:peiyuan_xia2013@163.com)

^†^ These authors contributed equally to this work.

**Additional files**

Additional figures including synthetic information and characterization results of CA-Oxi-αCD and its intermediate product, physicochemical properties of CA-Oxi-αCD NPs, IC_50_ of DTX and its nanoformulations on 4T1 and MDA-MB-231 cells, characterization of CA acetal, α-CD, CA-αCD and CA-Oxi-αCD, the evaluation of stability and pH/ROS responsiveness of CA-Oxi-αCD NPs, the biocompatibility of DTX loaded NPs, the DTX release profile from PLGA NPs in various medium, cellular uptake of MDA-MB-231 cells, the penetration ability of Cy5-labeled NPs in 4T1 tumor spheroids, the effect of NPs on 4T1 cell migration and invasion, the mitochondrial damage-induced apoptosis of MDA-MB-231 cells by DTX loaded NPs, *in vitro* antitumor effect of NPs on MDA-MB-231 cells, *in vivo* initial safety evaluation of DTX loaded NPs, immunofluorescence and immunohistochemistry examination of tumor tissue, representative photographs and weight of spleen tissue, the body weight and representative photographs of tumor tissues from mice after various treatment, representative photographs and weight of the spleen, *in vivo* initial safety evaluation of CA-Oxi-αCD NPs, H&E staining of the major organs from healthy mice with CA-Oxi-αCD NPs treatment.

**Reagents**

All chemical reagents and anhydrous solvents were obtained from commercial sources and used directly as received. 4-dimethylaminopyridine (DMAP), trimethoxymethane, α-Cyclodextrin (α-CD), N,N'-Carbonyldiimidazole (CDI), anhydrous dimethyl sulfoxide (DMSO) were purchased from Aladdin (Shanghai, China). Cinnamaldehyde, Pyridin-1-ium 4-methylbenzenesulfonate (PPTs) were purchased from J&K Scientific Co., Ltd. (Beijing, China). 4-Hydroxymethylphenylboronic acid pinacol ester was purchased from Sigma-Aldrich (St. Louis, MO, USA). Cy5 free acid and Cy5-NHS ester were purchased from Ruixi Biological Technology Co., Ltd. (Xi’an, China).

**Materials synthesis**

**Scheme S1**. Synthetic route of CA-Oxi-αCD.

**Synthesis of (E)-(3,3-dimethoxyprop-1-en-1-yl) benzene (1)**

Cinnamaldehyde (CA, 5.3762 g, 40.68 mmol ) and trimethoxymethane (19.40 g, 182 mmol ) were dissolved in methanol (50 mL), then Pyridin-1-ium 4-methylbenzenesulfonate (PPTs, 2.0421 g, 8.14 mmol) was added to the solution, such a mixture was refluxed at 60 °C for 3 h and monitored by thin layer chromatography with the mobile phase petroleum ether/ethyl acetate=20:1. After completion, the mixture was quenched with NaHCO_3_ saturated solution (20 mL) then extracted with ethyl acetate (40 mL) for three times, combining all the ethyl acetate and dried over MgSO_4_, the product CA acetal was given as yellow liquid by evaporated ethyl acetate using a rotary evaporator (6.8765 g). The reaction residue was used in the next step without further purification. These spectral data matched those previously reported [[1](#_ENREF_1)]. ^1^H NMR (600 MHz, DMSO-d6) δ 7.49 (d, J = 7.6 Hz, 2H), 7.35 (t, J = 7.6 Hz, 2H), 7.28 (t, J = 7.3 Hz, 1H), 6.69 (d, J = 16.2 Hz, 1H), 6.23 (dd, J = 16.2, 5.2 Hz, 1H), 4.94 (d, J = 5.2 Hz, 1H), 3.27 (s, 6H).

**Synthesis of** **CA-αCD (2)**

The α-Cyclodextrin (α-CD, 1.6238 g) and CA acetal (compound 1, 3.5696 g) were dissolved in anhydrous DMSO (14 mL), then Pyridin-1-ium 4-methylbenzenesulfonate (PPTs, 0.2512 g) was added to the mixture, the mixture was stirred for 72 h at 60 °C. After completion, the crude product was obtained by precipitated in acetone, followed by vacuum filtered, rinsed with acetone and dried at 80 °C to provide the desired product as light-yellow solid (CA-αCD, 2.1324 g).

**Synthesis of 4-(4,4,5,5-tetramethyl-1,3,2-dioxaborolan-2-yl)benzyl 1H-imidazole-1-carboxylate (3)**

4-Hydroxymethylphenylboronic acid pinacol ester (5.5324g, 24 mmol) was dissolved in anhydrous CH_2_Cl_2_ (36 mL), then N,N'-Carbonyldiimidazole (CDI, 7.6648 g, 48 mmol) was added to the mixture, the obtained reaction solution was submitted to stir at rt for 1 h. After completing the reaction, another CH_2_Cl_2_ (36 mL) was added to the reaction mixture, then the solution was washed with deionized water (30 mL) for three times, the organic phase was further washed with saturated NaCl solution (30 mL), dried over MgSO_4_. The desired product was provided through vacuum rotary evaporation as white solid (7.2346 g). ^1^H NMR (600 MHz, DMSO) δ 8.30 (s, 1H), 7.72 (d, J = 7.9 Hz, 2H), 7.62 (s, 1H), 7.50 (d, J = 7.8 Hz, 2H), 7.08 (s, 1H), 5.46 (s, 2H), 1.28 (s, 12H).

**Synthesis of CA-Oxi-αCD (4)**

The compound 4 was synthesized according to previous reference [[2](#_ENREF_2)]. Briefly, the obtained compound 2 (0.9946 g) was dissolved in anhydrous DMSO (30 mL), then DMAP (2.1991 g) was added, followed by the addition of CDI-activated 4-(hydroxymethyl) phenylboronic acid pinacol ester (compound 2, 1.1814 g), such mixture was stirred at 30 °C for 72 h. After the reaction accomplished, the crude product was obtained by precipitation from water (30 mL), then collected by centrifugation. After dialyzed against deionized water for 48 h, the sample was lyophilized to give off-white solid (CA-Oxi-αCD, 0.5764 g).

**Characterization**

^1^H Nuclear Magnetic Resonance (^1^H NMR) data was collected at 25 °C on a 600 MHz spectrometer (Direct Drive 2, Agilent) and chemical shifts were reported relative to tetramethylsilane (TMS, δ = 0). Fourier-transform infrared (FT-IR) spectra was performed on a PerkinElmer FT-IR spectrometer (100S, U.S.A.).

**Reference**

[1] C. Zhao, W. Cao, H. Zheng, Z. Xiao, J. Hu, L. Yang, M. Chen, G. Liang, S. Zheng, C. Zhao, *Int. J. Nanomed.* **2019**, 14, 1597.

[2] D. Zhang, Y. Wei, K. Chen, X. Zhang, X. Xu, Q. Shi, S. Han, X. Chen, H. Gong, X. Li, J. Zhang, *Adv. Healthc. Mater.* **2015**, 4, 69.

**Table S1**. Physicochemical properties of various NPs.

| Nanoformulations | Size (nm) | PDI | Zeta potential (mV) | Drug loading (w/w %) | Encapsulation efficiency (%) |
| --- | --- | --- | --- | --- | --- |
| Blank FA-CA-Oxi-αCD NPs | 206.2 ± 1.4 | 0.11 ± 0.04 | -18.6 ± 0.6 | - | - |
| DTX/CA-Oxi-αCD NPs | 213.9 ± 4.8 | 0.06 ± 0.05 | -18.5 ± 0.3 | 16.95 ± 3.07 | 59.34 ± 5.40 |
| DTX/FA-CA-Oxi-αCD NPs | 227.9 ± 0.8 | 0.16 ± 0.02 | -18.8 ± 0.2 | 21.53 ± 3.12 | 75.98 ± 6.90 |
| DTX/PLGA NPs | 240.7 ± 9.1 | 0.21 ± 0.08 | -23.0 ± 2.35 | 34.80 ± 0.04 | 87.26 ± 0.04 |
| Cy5-CA-Oxi-αCD NPs | 182.8 ± 6.5 | 0.19 ± 0.06 | -17.5 ± 0.9 | - | - |
| Cy5-FA-CA-Oxi-αCD NPs | 194.9 ± 3.9 | 0.12 ± 0.01 | -18.0 ± 1.3 | - | - |

**Table S2**. IC_50_ of DTX and its nanoformulations on 4T1 and MDA-MB-231 cells.

| IC_50_  ng/mL | DTX | DTX/  CA-Oxi-αCD NPs | DTX/FA-  CA-Oxi-αCD NPs |
| --- | --- | --- | --- |
| 4T1 | 6.79 | 1.97 | 0.69 |
| MDA-MB-231 | 68.16 | 59.05 | 31.69 |


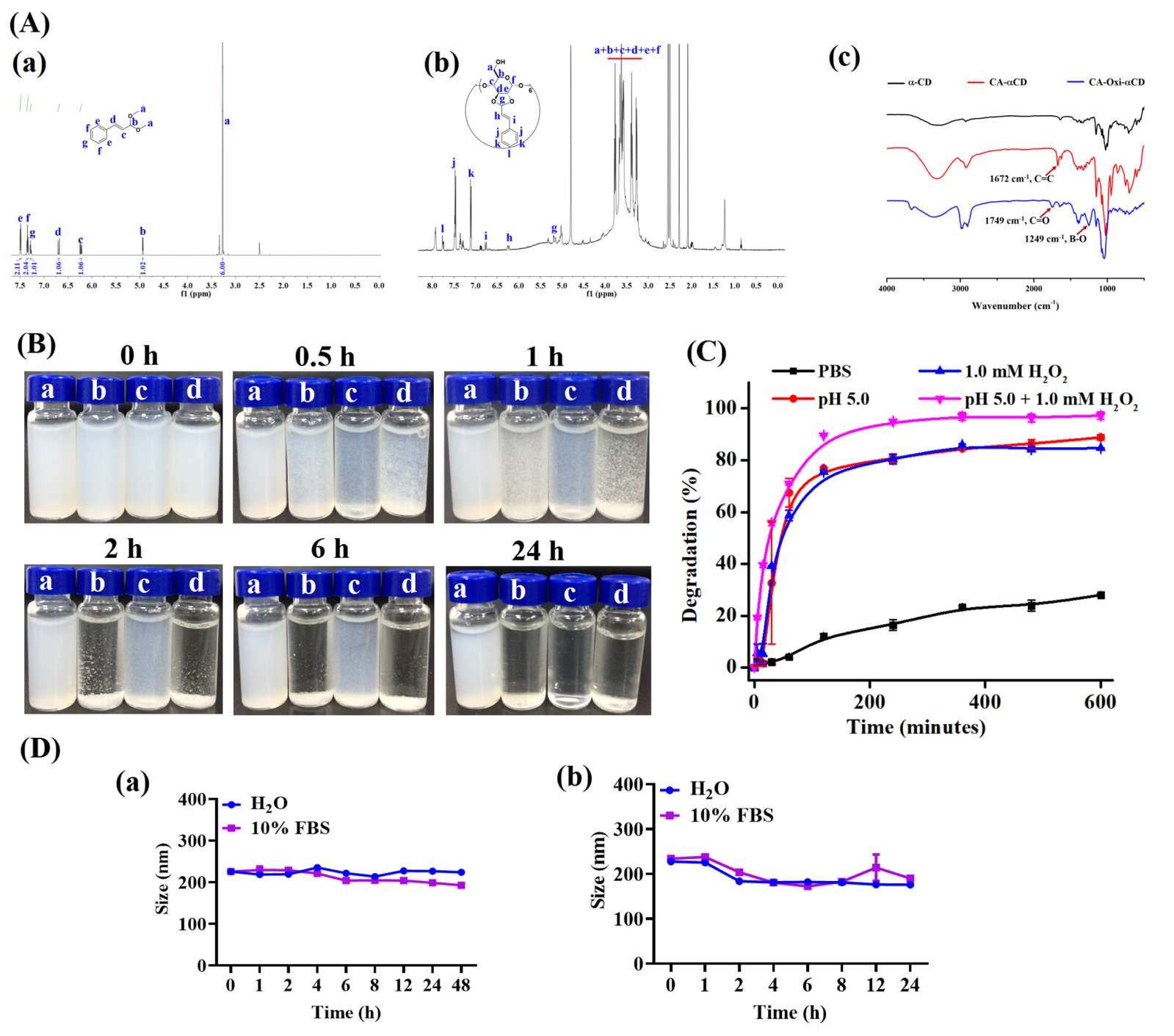


**Figure S1**. The characterization of CA acetal, α-CD, CA-αCD and CA-Oxi-αCD and evaluation of stability and pH/ROS responsiveness of CA-Oxi-αCD NPs. (A) (a) ^1^H NMR spectrum of CA acetal in DMSO-d_6_. (b) ^1^H NMR spectrum of CA-αCD in DMSO-d_6_. (c) Infrared spectra of α-CD, CA-αCD and CA-Oxi-αCD. (B) The photo images of pH/ROS-triggered disassembly of Blank CA-Oxi-αCD NPs. a-d, with the incubation of PBS, 1 mM H_2_O_2_ in PBS, PBS at pH 5.0 or 1 mM H_2_O_2_ in PBS at pH 5.0, respectively. (C) The quantitative analysis of degradation of Blank CA-Oxi-αCD NPs with the various medium incubation. (D) (a) The size distribution of DTX/CA-Oxi-αCD NPs and (b) DTX/FA-CA-Oxi-αCD NPs in H_2_O or 10 % FBS.


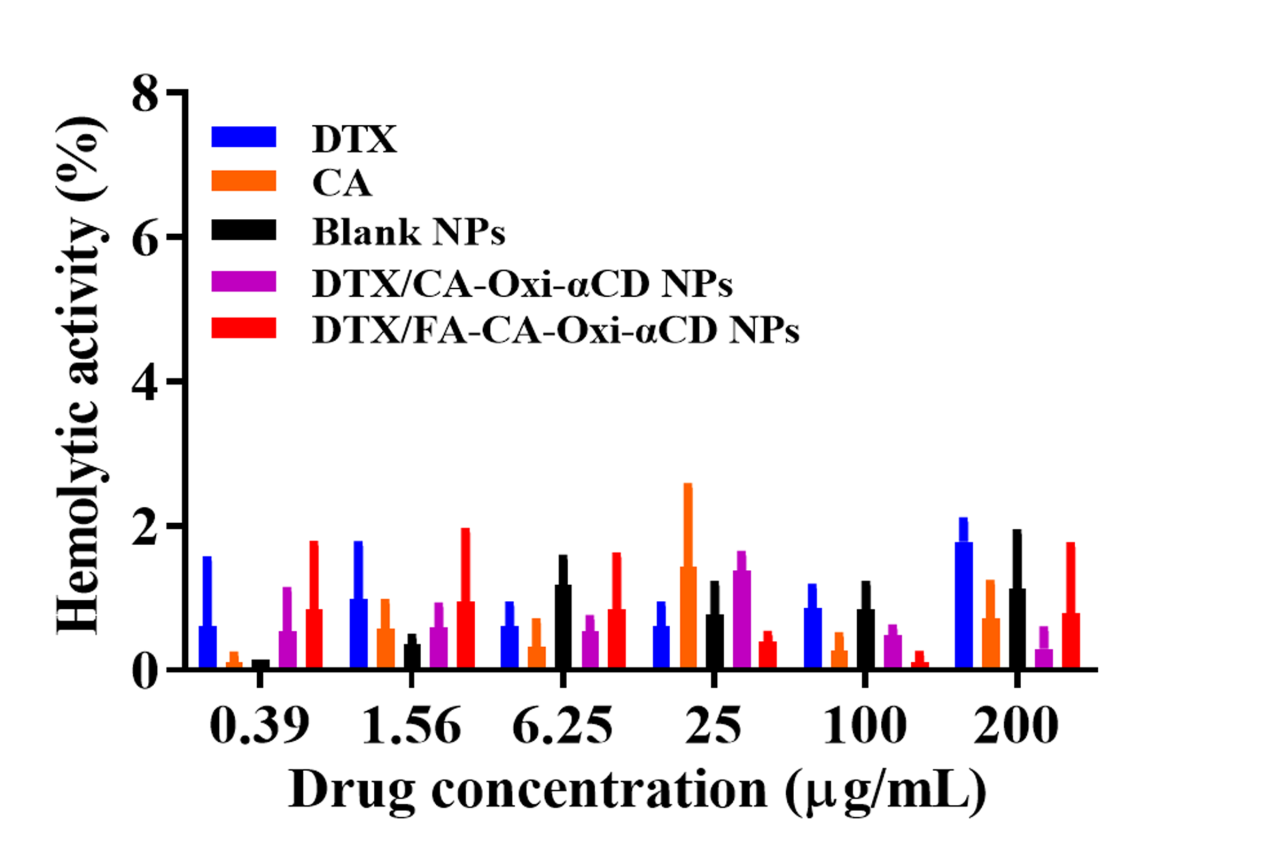


**Figure S2**. The biocompatibility of various drugs with incubation of sheep blood.


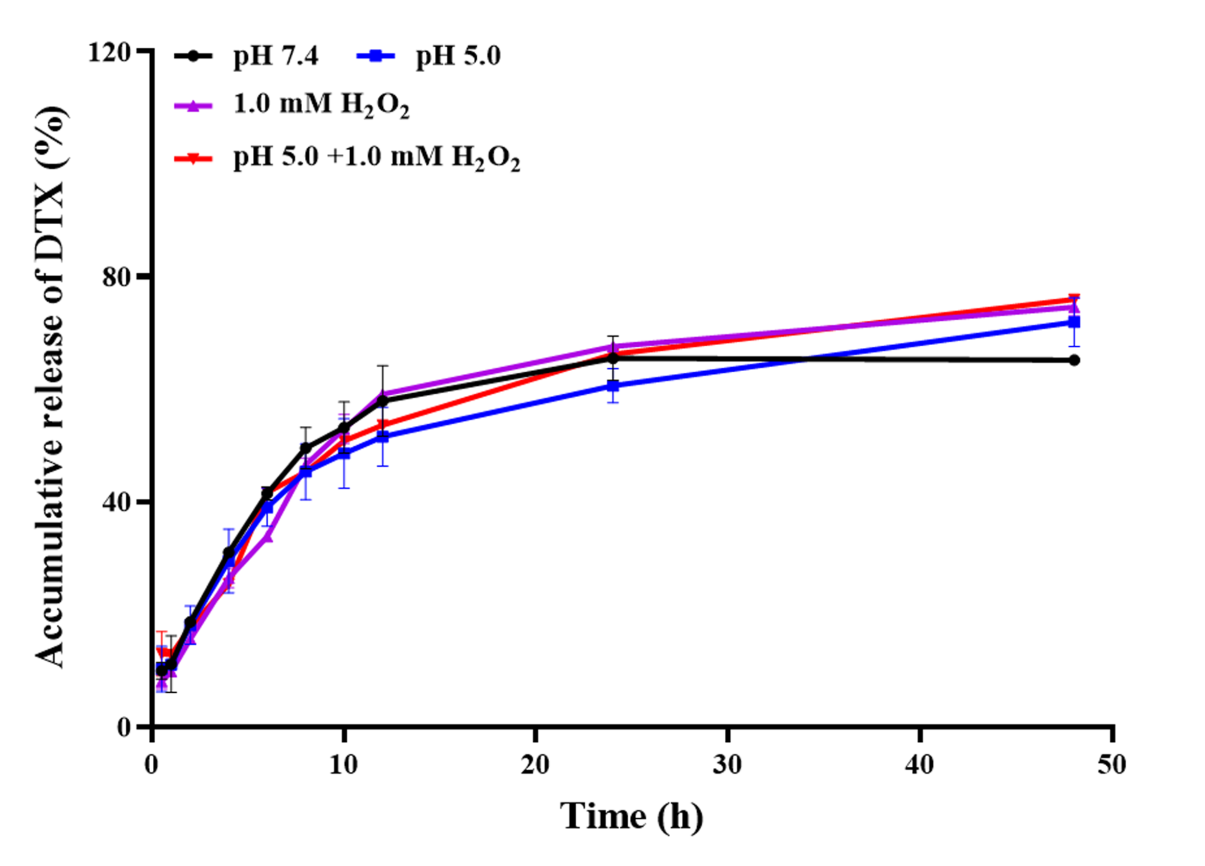


**Figure S3**. The DTX release profile from DTX/PLGA NPs in various medium. Data represent mean ± SD (n = 3).


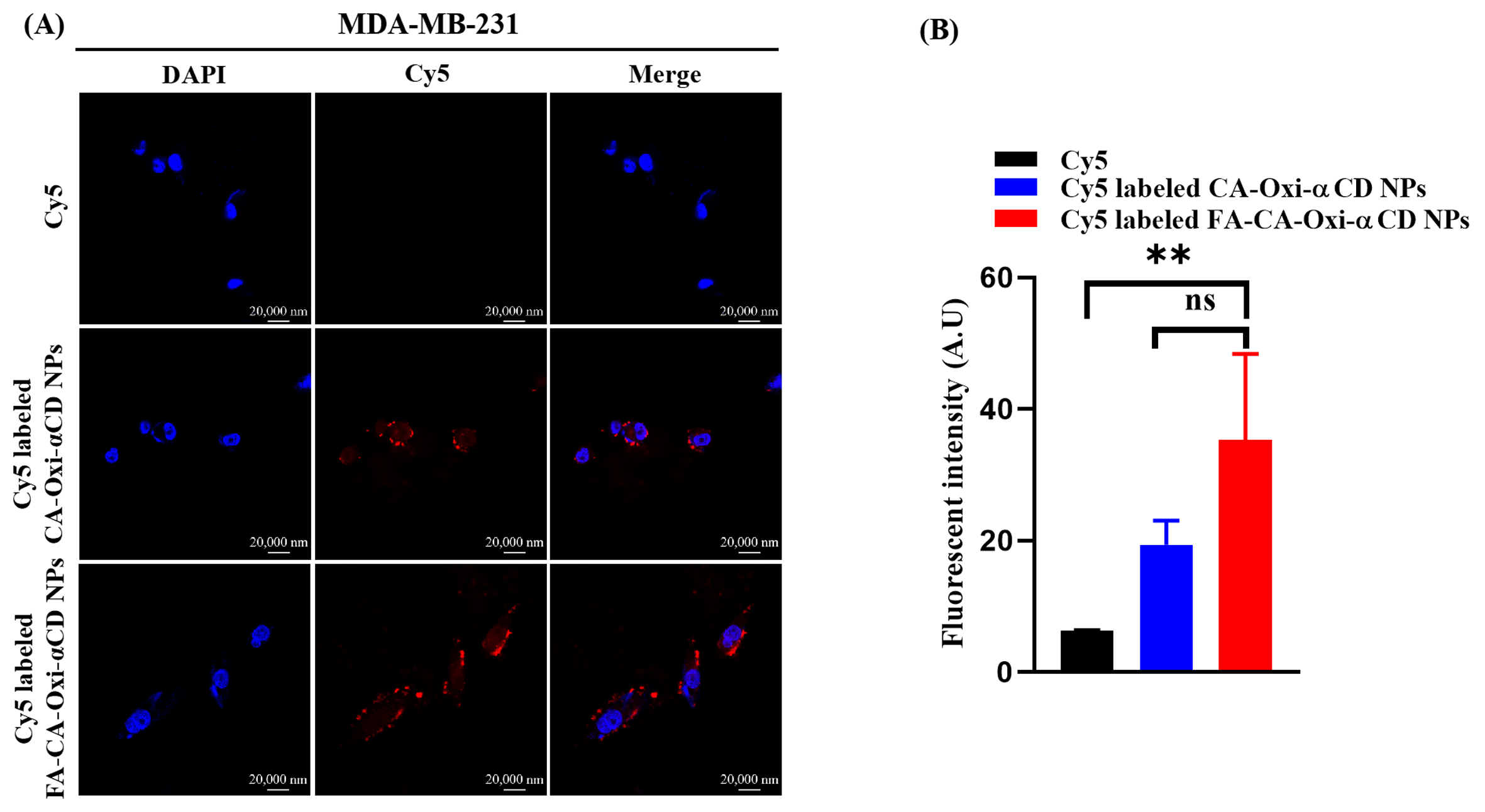


**Figure S4**. (A) CLSM images of cellular uptake of MDA-MB-231 cells treated with Cy5, Cy5-labeled CA-Oxi-αCD NPs and Cy5-labeled FA-CA-Oxi-αCD NPs for 6 h. DAPI for nuclei staining (blue), Cy5 labeled NPs (red). Scale bar represents 20 μm. (B) The semi-quantitative analysis of the corresponding Cy5 fluorescence intensity of intracellular NPs (red) in MDA-MB-231 cells. **p*<0.05, ***p*<0.01, ****p*<0.001, ns, no significant difference, compared with Cy5 labeled FA-CA-Oxi-αCD NPs (n=3).


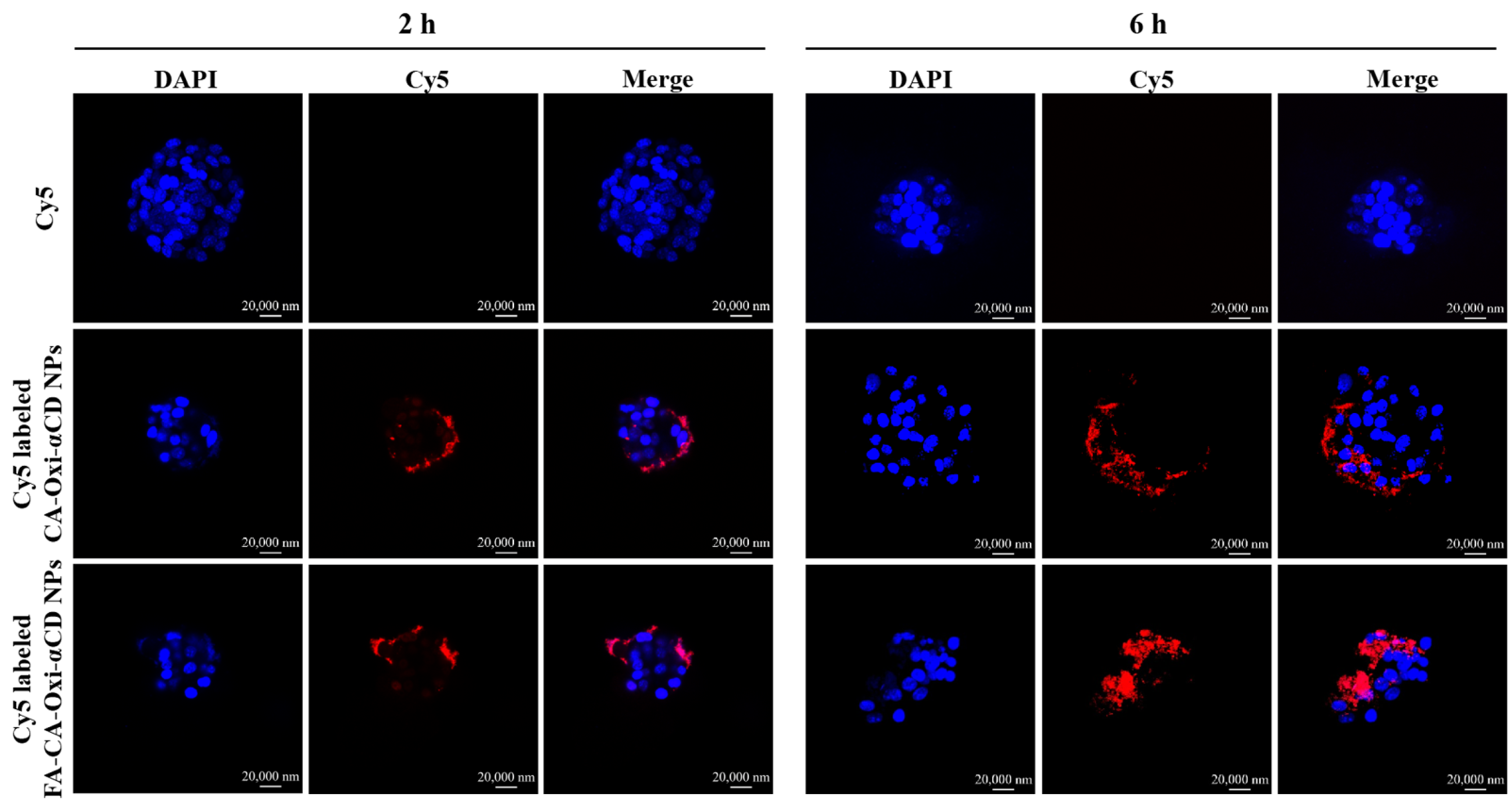


**Figure S5**. The penetration ability of Cy5-labeled NPs in 4T1 tumor spheroids. Fluorescence distribution of Cy5-labeled NPs in tumor spheroid sections were observed at 2 h and 6 h by CLSM. Cell nuclei were stained with DAPI (blue), Cy5 labeled NPs (red). Scale bar represents 20 μm.


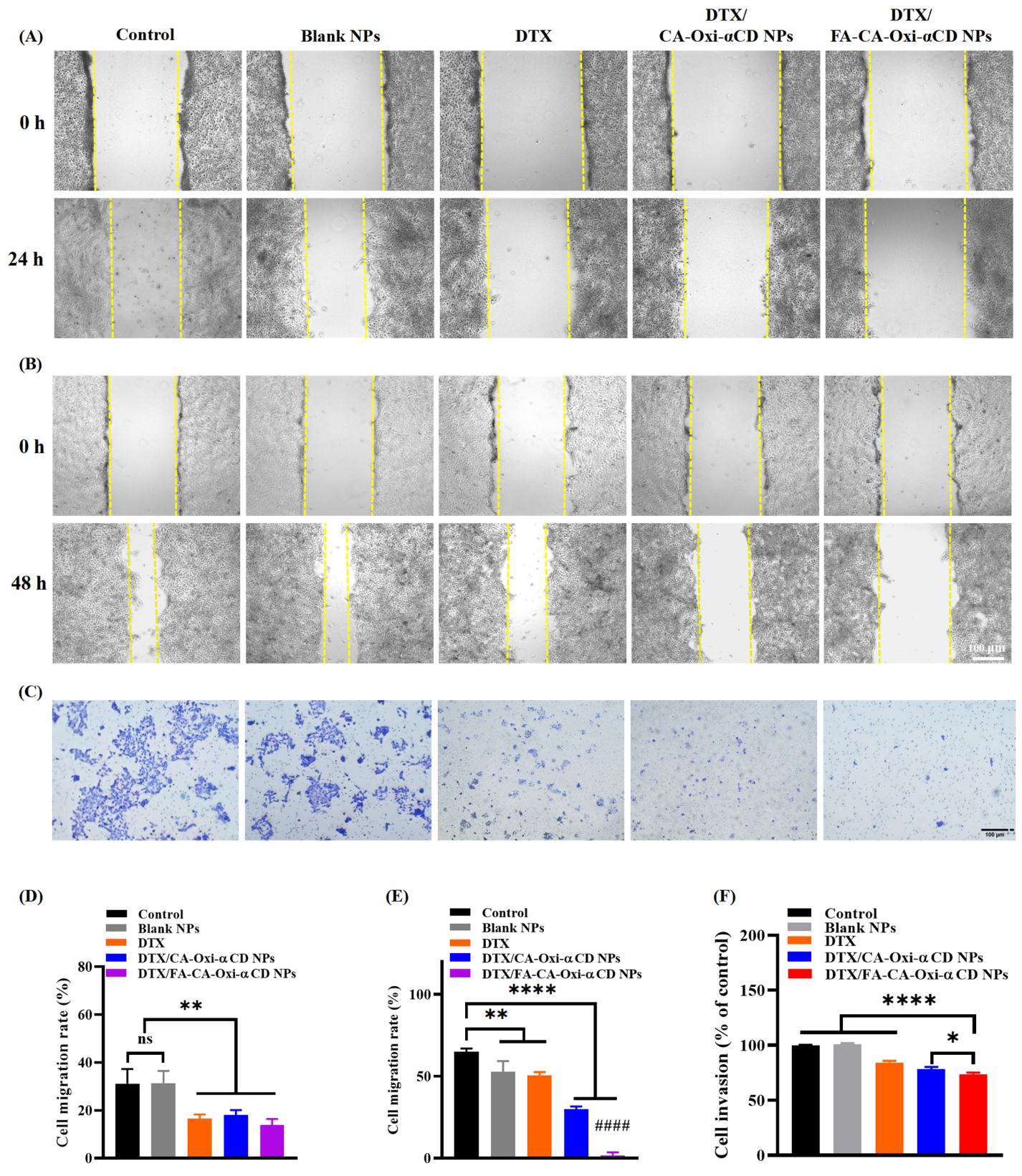


**Figure S6**. The effect of NPs on 4T1 cell migration and invasion. (A, B) The wound healing images of 4T1 cells after treated with Blank NPs, DTX/CA-Oxi-αCD NPs and DTX/FA-CA-Oxi-αCD NPs at 0 h, 24 h and 48 h. Scale bar represents 100 μm. (C) Microscopy images of 4T1 cells migrated and attached to the underside of Transwell membrane after being stained with 0.1% crystal violet. Scale bar represents 100 μm. (D, E) The quantitative analysis of cell migration rate of 4T1 cells after different treatment for 24 h and 48 h. **p*<0.05, ***p*<0.01, ****p*<0.001, *****p*<0.0001, ns, no significant difference, compared with control. ^#^*p*<0.05, ^##^*p*<0.01, ^###^*p*<0.001, ^####^*p*<0.0001, compared with DTX/FA-CA-Oxi-αCD NPs (n=3). (F) The quantitative analysis of cell invasion percentages of 4T1 cells after different treatment for 48 h. **p*<0.05, ***p*<0.01, ****p*<0.001, *****p*<0.0001, compared with DTX/FA-CA-Oxi-αCD NPs (n=3).


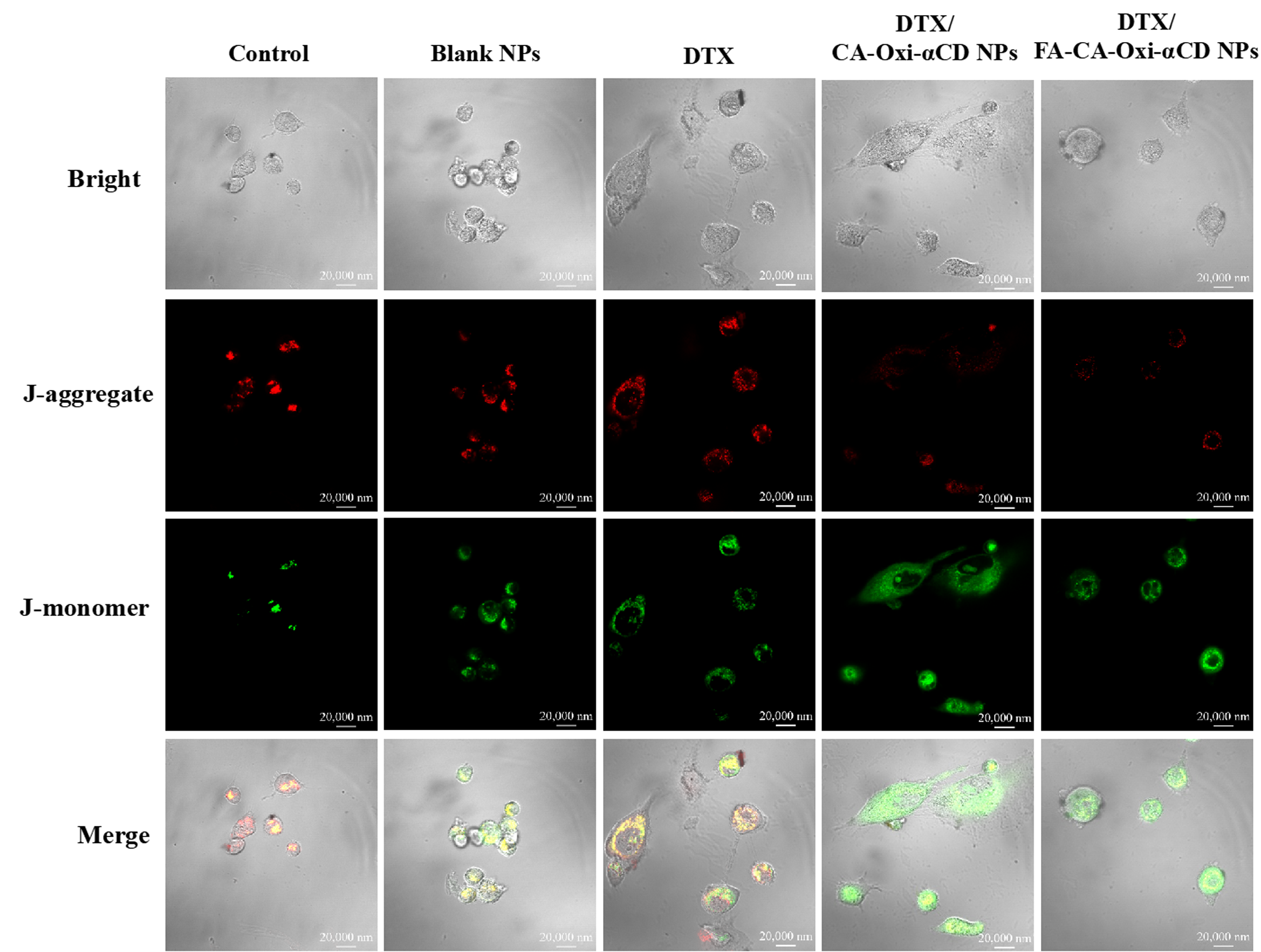


**Figure S7**. Mitochondrial damage-induced apoptosis of MDA-MB-231 cells by DTX loaded NPs. CLSM images of JC-1 stained cells after different treatments, the fluorescence transition from red to green indicated significant mitochondrial damage. Scale bar represents 20 μm.


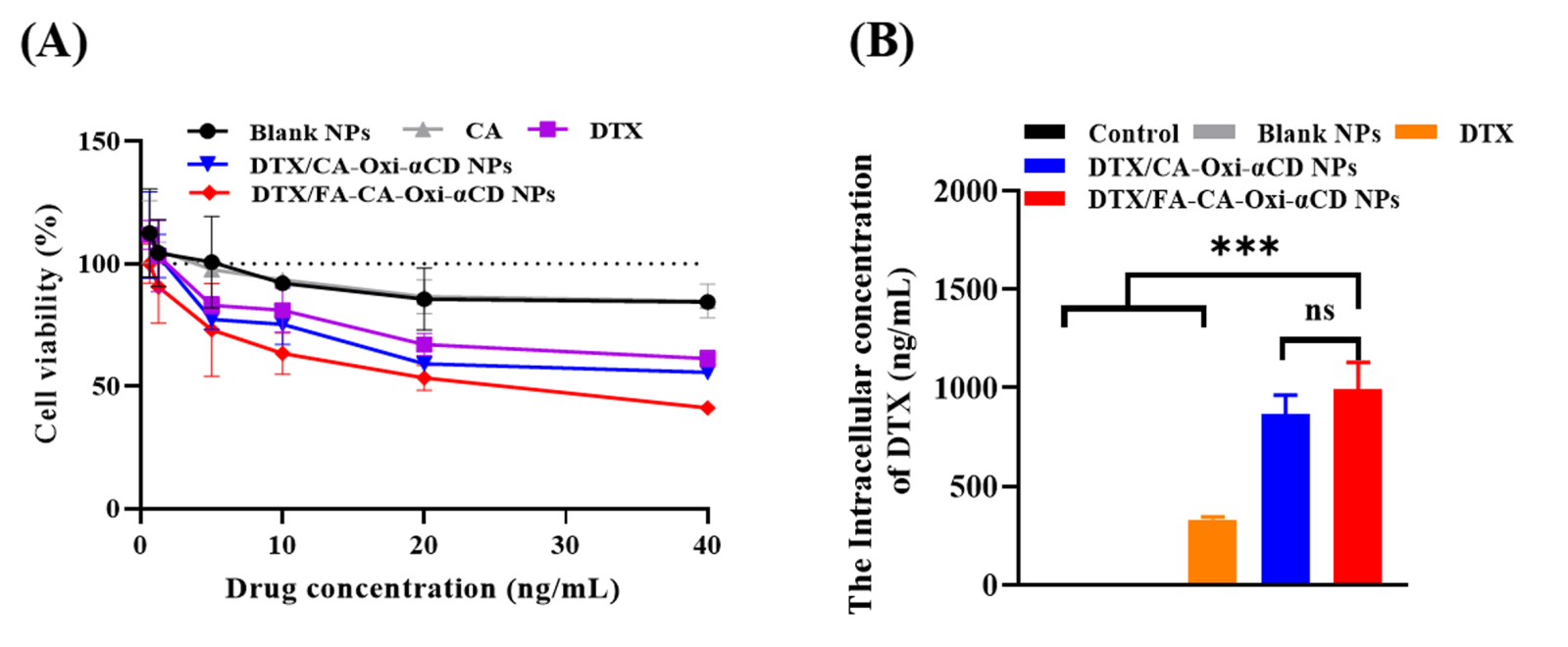


**Figure S8**. The *in vitro* antitumor effect of NPs on MDA-MB-231 cells. (A) Cell viabilities of MDA-MB-231 cells after treatment with DTX, CA, Blank NPs, DTX/CA-Oxi-αCD NPs and DTX/FA-CA-Oxi-αCD NPs with various drug concentration for 48 h. (B) The intracellular concentration of DTX in MDA-MB-231 cells treated with DTX, Blank NPs, DTX/CA-Oxi-αCD NPs and DTX/FA-CA-Oxi-αCD NPs for 48 h. The controls were treated with cell culture medium. **p*<0.05, ***p*<0.01, ****p*<0.001, and ns, no significant difference, compared with FA-CA-Oxi-αCD NPs (n=3).


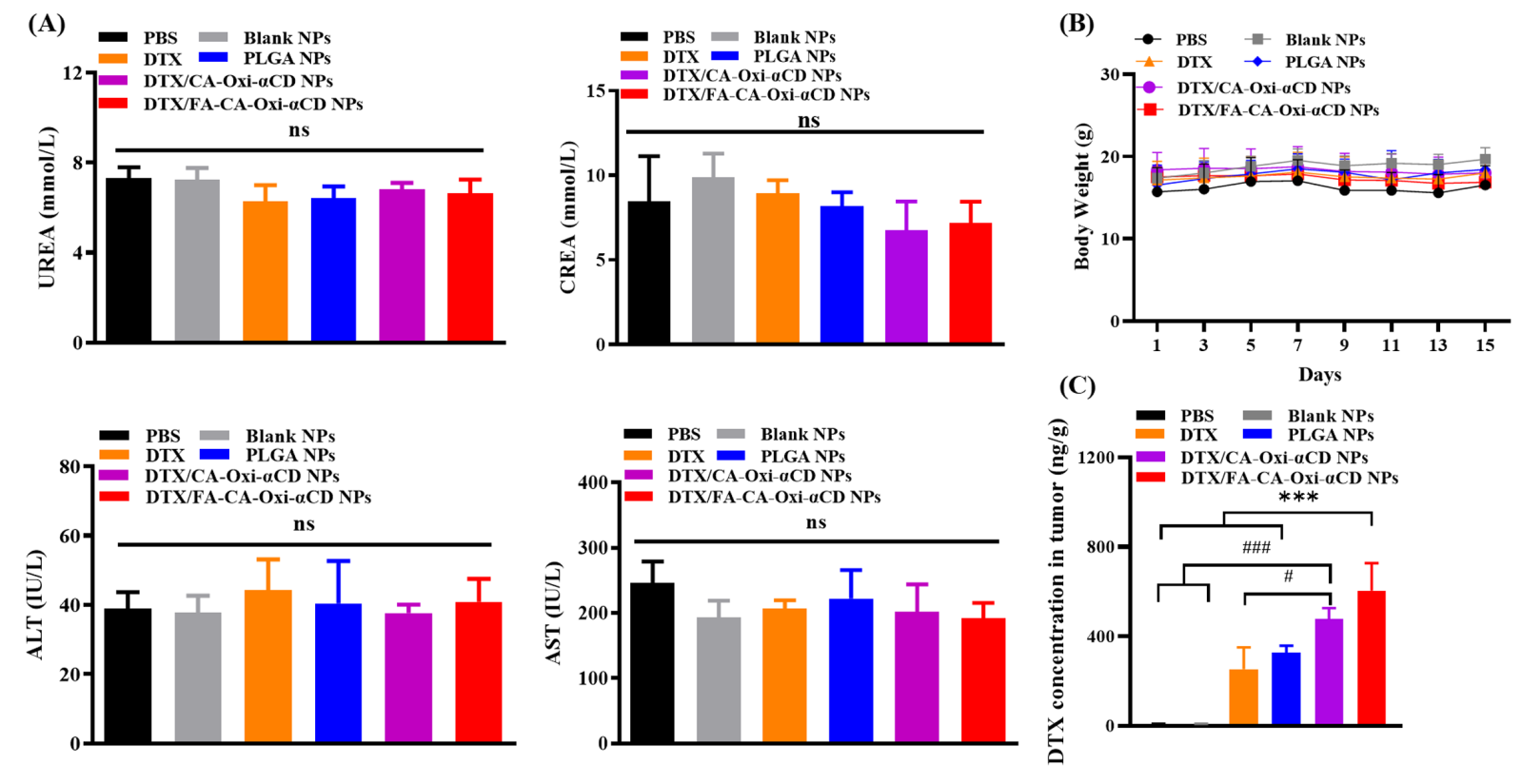


**Figure S9**. *In vivo* safety evaluation and the DTX distribution in tumors from mice with PBS, DTX, Blank NPs, PLGA NPs, DTX/CA-Oxi-αCD NPs and DTX/FA-CA-Oxi-αCD NPs treatment. (A) The levels of liver enzymes and the concentration of markers related to renal function. ALT, alanine aminotransferase; AST, aspartate aminotransferase. UREA, urea; CREA, creatinine. (B) The body weight of mice after *i.v.* administration with PBS, DTX, Blank NPs, PLGA NPs, DTX/CA-Oxi-αCD NPs and DTX/FA-CA-Oxi-αCD NPs. (C) The DTX concentration in tumors from mice with free DTX and various NPs treatment. **p*<0.05, ***p*<0.01, and ****p*<0.001, ns, no significant difference, compared with DTX/FA-CA-Oxi-αCD NPs. ^#^*p*<0.05, ^##^*p*<0.01, ^###^*p*<0.001, compared with DTX/CA-Oxi-αCD NPs.


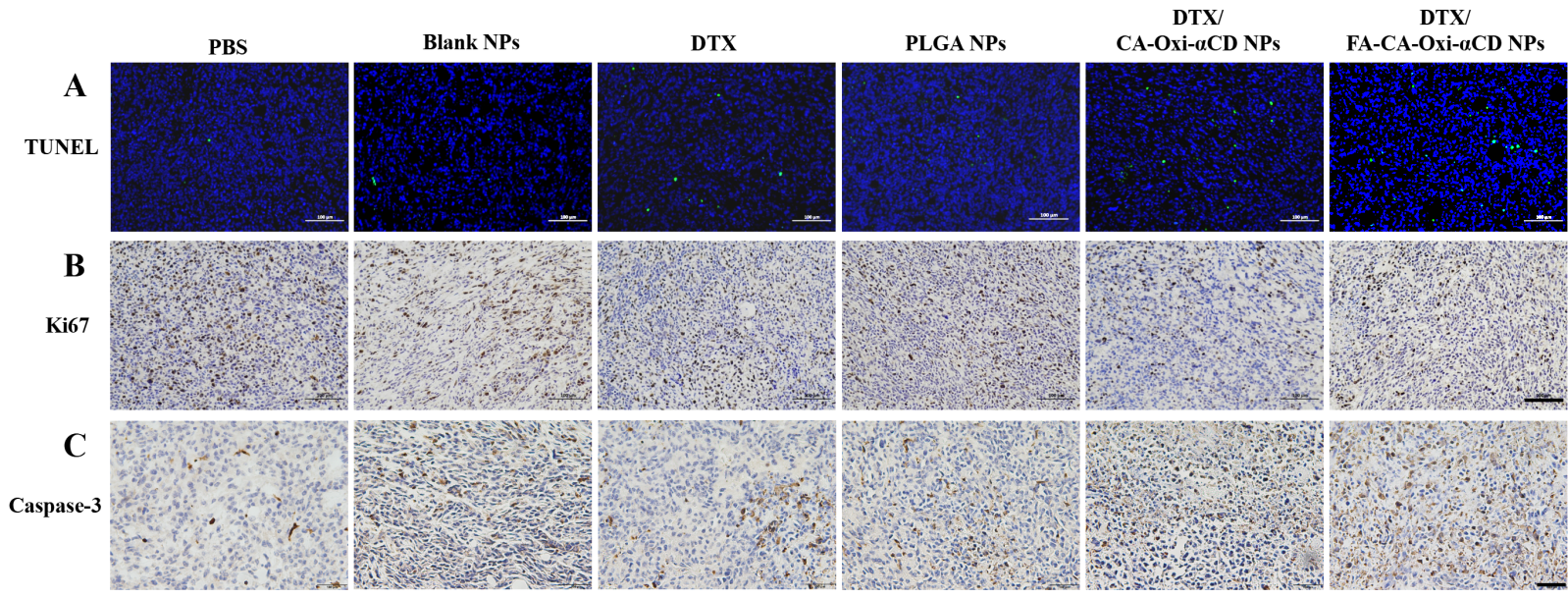


**Figure S10**. Immunofluorescence and immunohistochemistry examination of tumor tissue from mice with free DTX and various NPs treatment. (A) Immunofluorescence staining images of tumor tissue in different groups. DAPI for nuclei staining (blue), and TUNEL staining (green) were observed. Scale bar represents 100 μm. (B) Immunohistochemistry assay for Ki67 in tumor tissues. Scale bar represents 100 μm. (C) Immunohistochemistry assay for caspase-3 in tumor tissues. Scale bar represents 100 μm.


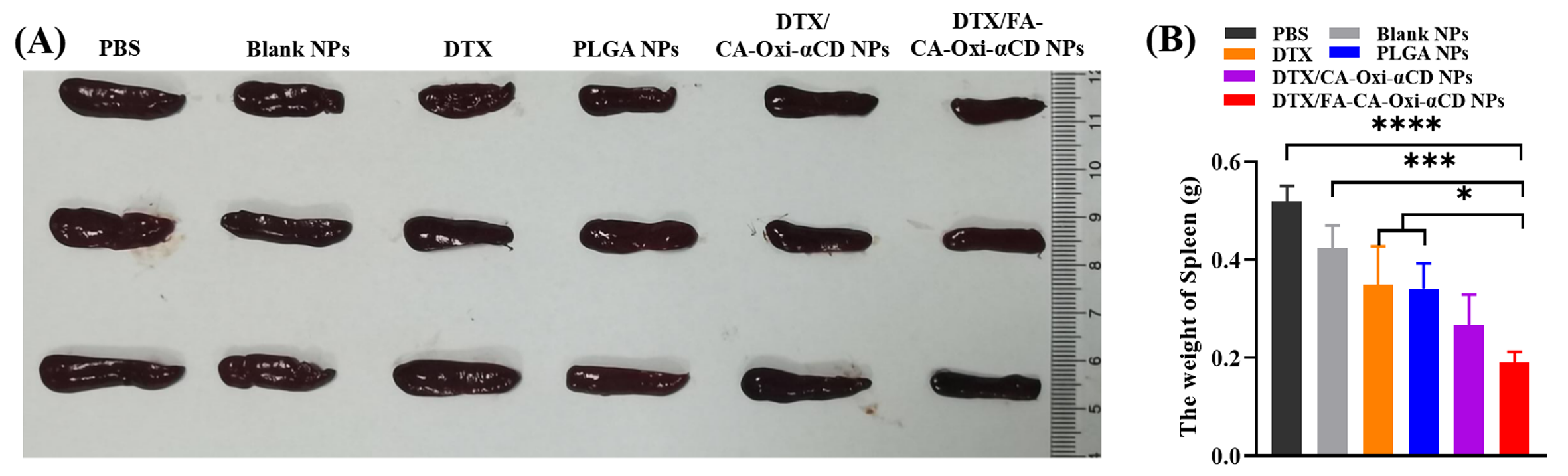


**Figure S11**. (A) Representative photographs of spleen tissue from mice after treatment with PBS, DTX, Blank NPs, PLGA NPs, DTX/CA-Oxi-αCD NPs and DTX/FA-CA-Oxi-αCD NPs, respectively. (B) The spleen weight of mice with the treatment of free DTX and various NPs. **p*<0.05, ***p*<0.01, and ****p*<0.001, *****p*<0.0001, compared with DTX/FA-CA-Oxi-αCD NPs (n=3).


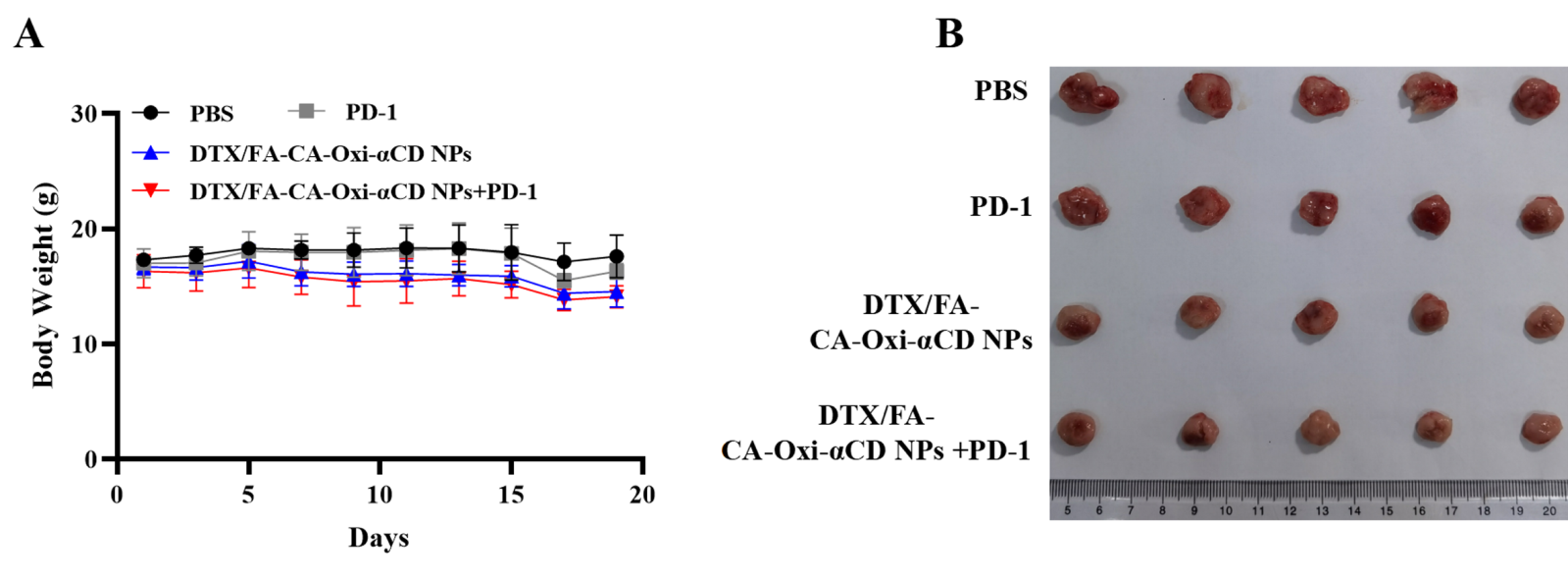


**Figure S12**. (A) The body weight after *i.v.* administration with PBS, *i.p.* administration with anti-PD-1 antibody, *i.v.* administration with DTX/FA-CA-Oxi-αCD NPs and DTX/FA-CA-Oxi-αCD NPs with anti-PD-1 antibody, respectively. (B) Representative photographs of tumor tissues from mice after various treatment.


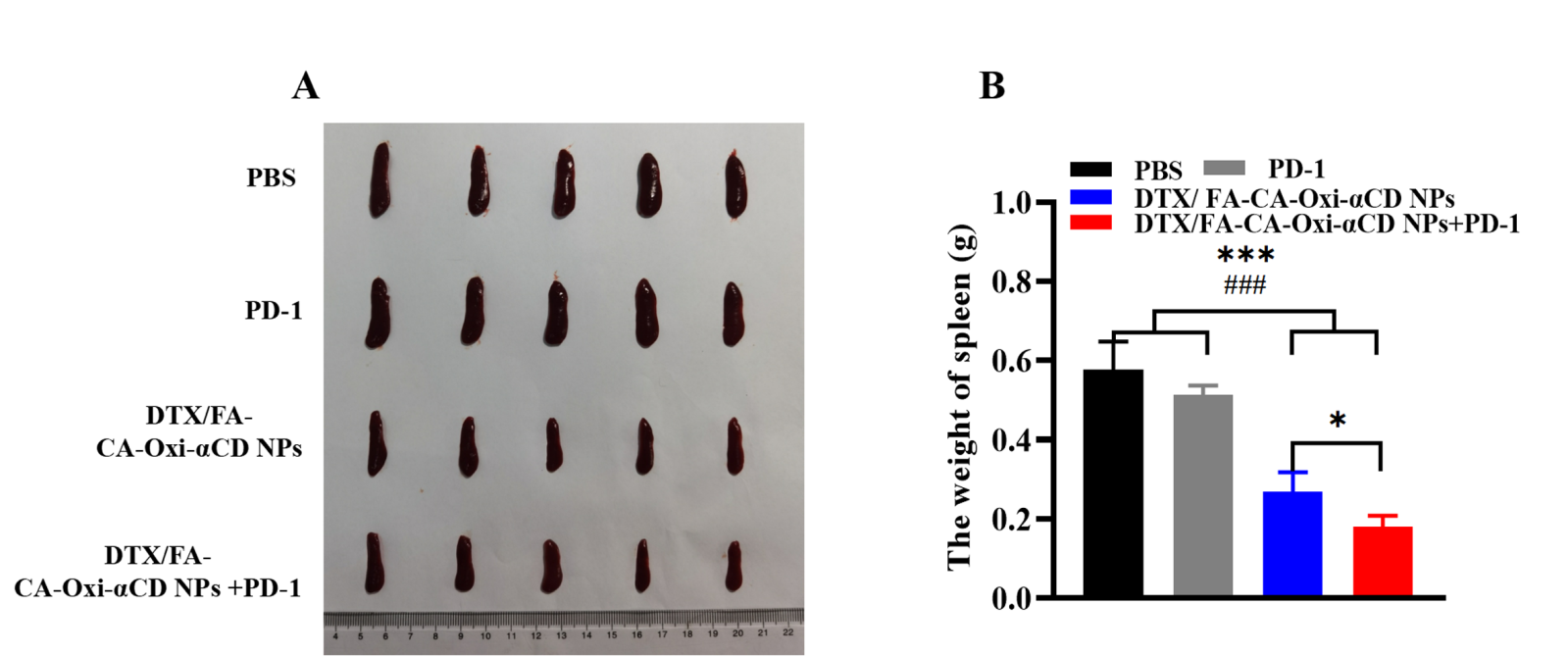


**Figure S13**. (A) Representative photographs of spleen from mice after various treatment. (B) The spleen weight of mice in different treatment groups. **p*<0.05, ***p*<0.01, ****p*<0.001, compared with DTX/FA-CA-Oxi-αCD NPs + PD-1, ^#^*p*<0.05, ^##^*p*<0.01, ^###^ *p*<0.001, compared with DTX/FA-CA-Oxi-αCD NPs (n=3).


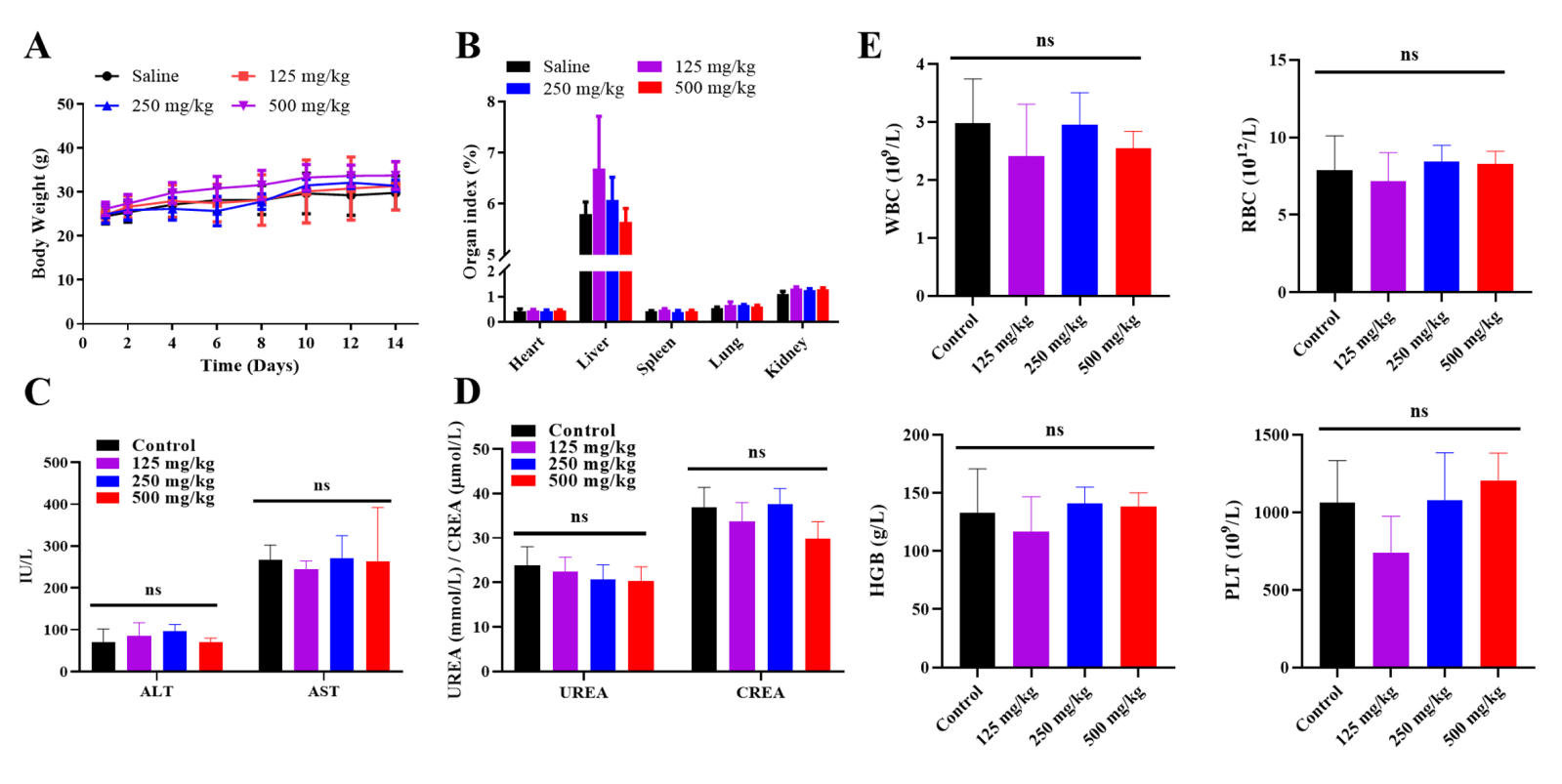


**Figure S14**. *In vivo* initial safety evaluation of CA-Oxi-αCD NPs. (A) The body weight of mice after *i.v.* administration with CA-Oxi-αCD NPs at various doses. (B) The organ index of major tissues of mice in various groups. (C) The levels of liver enzymes. ALT, alanine aminotransferase; AST, aspartate aminotransferase. (D) The concentration of markers related to renal function. UREA, urea. CREA, creatinine. (E) Hematological parameters of blood samples from mice in various groups. WBC, white blood cell. RBC, red blood cell. HGB, hemoglobin and PLT, platelet. The control was treated with saline. ns, no significant difference. Data are mean ± SD (n = 4).


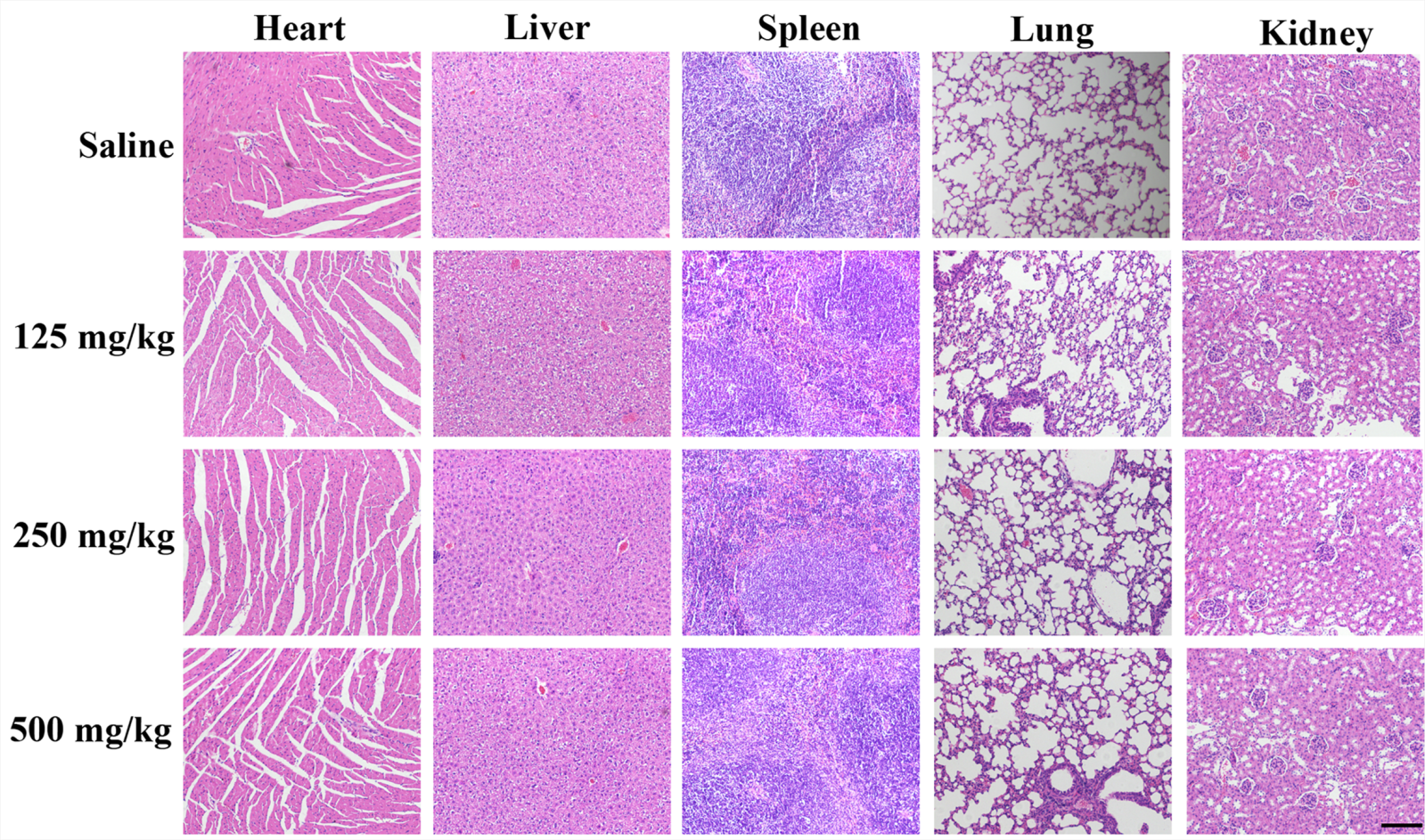


**Figure S15**. H&E staining of the major organs from healthy mice with CA-Oxi-αCD NPs treatment at the dose of 125 mg /kg, 250 mg/kg and 500 mg/kg. Scale bar represents 100 μm.
